# Supplementary material for: Identifying vaccine escape sites via statistical comparisons of short-term molecular dynamics
Source: Biophys Rep (N Y). 2022 Apr 4;2(2):100056. doi: 10.1016/j.bpr.2022.100056 (PMC8978532; doi:10.1016/j.bpr.2022.100056)
Supplement: Document S1. Figures S1–S4 and Table S1 [file mmc1.pdf]

Biophysical Reports, Volume *2*

Supplemental information

Identifying vaccine escape sites via statistical comparisons of short-term molecular dynamics

Madhusudan Rajendran, Maureen C. Ferran, and Gregory A. Babbitt

A

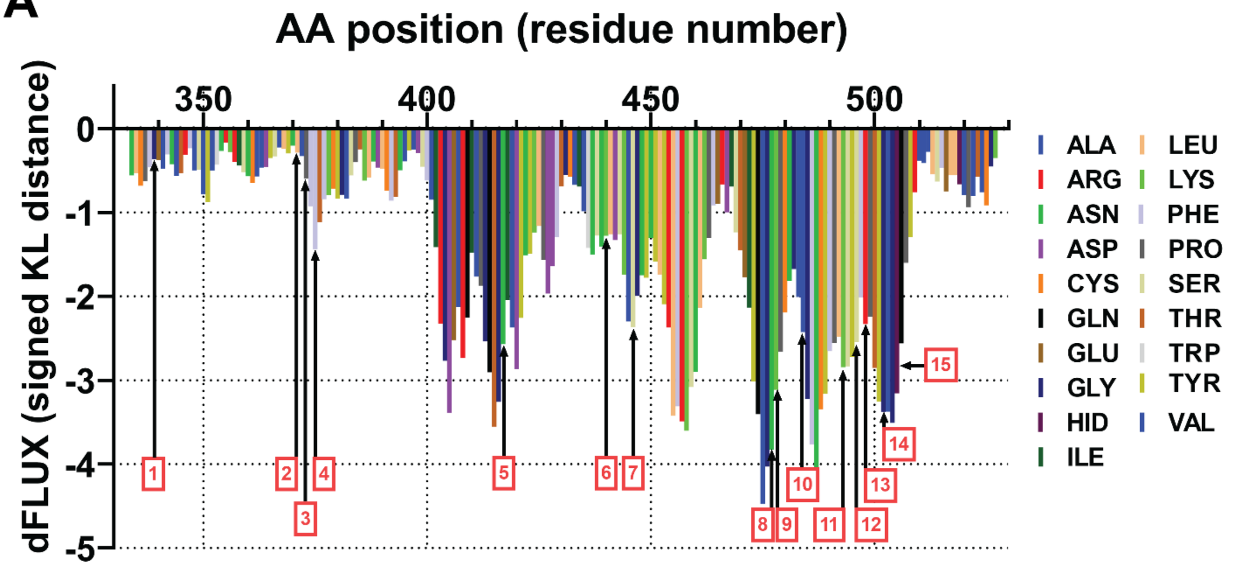

B

PDB ID	Antigen	Antibody	AA and position	-KL divergence	D value	p value
**7NXA**	Omicron RBD	COVOX-222	K417N (5)	-2.60	0.631	p < 0.001
			S477N (8)	-3.83	0.762	p < 0.001
			Q493K (11)	-2.84	0.762	p < 0.001
			N501Y (14)	-3.25	0.737	p < 0.001
			Y505H (15)	-3.16	0.771	p < 0.001

Supplemental Figure 1: Molecular dynamic simulation of the omicron variant. (A) Sequence positional plotting of dampening of atom motion on omicron RBD domain by COVOX-222. Mutation sites correspond to the Omicron RBD are labeled in red (1-15) and are listed in Figure 4B. Table summarizing the protein structure used for primary models for analyzing the molecular dynamics of COVOX-222 interaction with Omicron RBD. The table also includes amino acid positions, the corresponding -KL divergence value denoting atomic fluctuations dampening, D value and the corresponding level of significance for the KL divergence values.

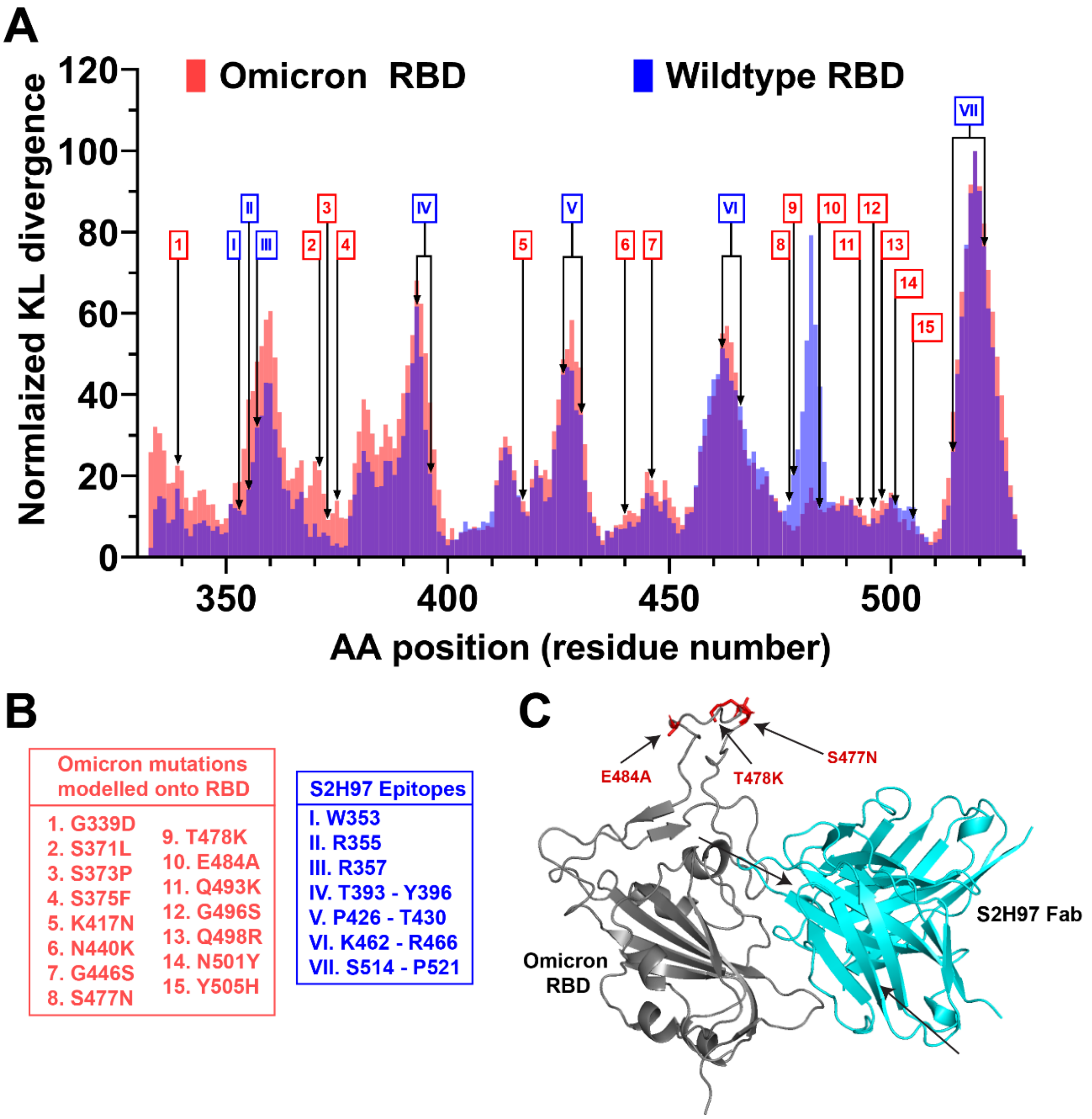

Supplemental Figure 2: MD simulations with the Omicron variant reveal sites that promote similiar binding affinity to hACE2. (A) Sequence positional plotting of the normalized dampening of atom motions on the Omicron RBD (red) and the wildtype RBD (blue) by monoclonal antibody S2H97.. The omicron RBD mutations are labeled in red (1-15). The sites corresponding to the S2H97 epitope on the wildtype RBD on labeled in blue (I – VII). The amino acid residues that correspond to the numbers and roman numerals are listed in (B). In the omicron RBD, we see alteration in atomic fluctuation peaks at several of the epitope sites.. (B) List of omicron mutations modeled onto RBD, and the list of S2H97 epitopes. (C) Crystal structure of S2H97 Fab (cyan) superimposed onto the structure of the Omicron RBD (grey) (PDB 7M7W). Some of the amino acid residues that correspond to the S2H97 epitopes, show an increase in atomic fluctuation dampening, are shown in blue. Several of the amino acid residue that correspond to omicron mutations with increased atomic fluctuation dampening, which are sites of ACE2 interactions, are shown in red. NOTE: Major changes occurring E4848A, T478K, S477N.

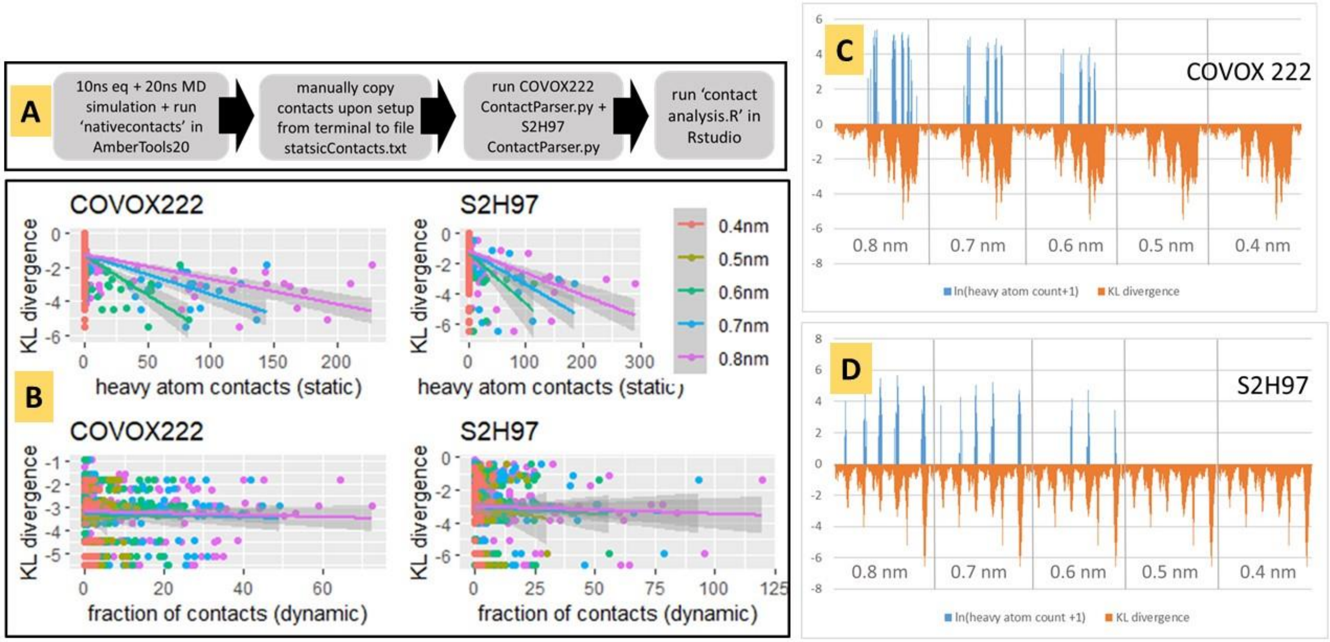

Supplemental Figure 3: Correlation between counts of heavy atom contacts between protein sites in the interface and atom dampening of sites during binding (i.e. KL divergence). The trends are shown for all interface sites for both COVOX-222 and S2H97 that fall within 4, 5, 6, 7, and 8 angstroms proximity range. Heavy atom contacts were counted in both static and dynamic conditions. For dynamic counts 100000 frames were generated from 20ns of molecular dynamics simulations on the antibody-bound viral receptor binding domain. For static counts only the first frame was used. All supporting data and code are in the S3 folder in our supplemental data file (data_RajendranFerranBabbitt_2022) (A) The analysis pipeline begins with the 'nativecontacts' program in AmberTools20 (see README.txt for the bash commands used) and proceeds subsequently via our python and R scripts (COVOX222_ContactsParser.py, S2H97_ContactParser.py, contact_analysis.R). (B) The associations between static and dynamic contact counts and the KL divergence are shown for each (See also Supplemental Table 1 below). (C-D) The respective positional plots of heavy atom contact counts and KL divergence are given for cutoff distance values of 0.4 nm – 0.8 nm. NOTE: the KL divergence mapping does not change with cutoff value and the heavy atom contact counts on the plots are scaled to ln(count+1) to allow visual comparison.

Supplemental Table 1. Correlation analyses for heavy atom contact counts and atom motion dampening (i.e. KL divergence) in SARS-CoV-2 antibody bound structures and molecular dynamic simulations of COVOX 222 and S2H97.

Correlation COVOX222	r (static)	t (static)	p (static)	r (dynamic)	t (dynamic)	p (dynamic)
0.8 nm	-0.5182787	-8.4192	8.48e-15	-0.0395004	-0.97635	0.3293
0.7 nm	-0.496611	-7.9486	1.541e-13	-0.039234	-0.79018	0.4299
0.6 nm	-0.458811	-7.1736	1.524e-11	-0.025573	-0.43788	0.6618
0.5 nm	NA	NA	NA	0.061458	0.60956	0.5436
0.4 nm	NA	NA	NA	0.0045329	0.044874	0.9643

correlation S2H97	r (static)	t (static)	p (static)	r (dynamic)	t (dynamic)	p (dynamic)
0.8 nm	-0.4745286	-7.5473	1.635e-12	-0.044418	-0.89479	0.3714
0.7 nm	-0.4501416	-7.0574	2.861e-11	-0.030993	-0.53976	0.5898
0.6 nm	-0.2893524	-4.232	3.556e-05	-0.052133	-0.74012	0.4601
0.5 nm	NA	NA	NA	-0.079197	-0.78648	0.4335
0.4 nm	NA	NA	NA	0.093881	0.9335	0.3529

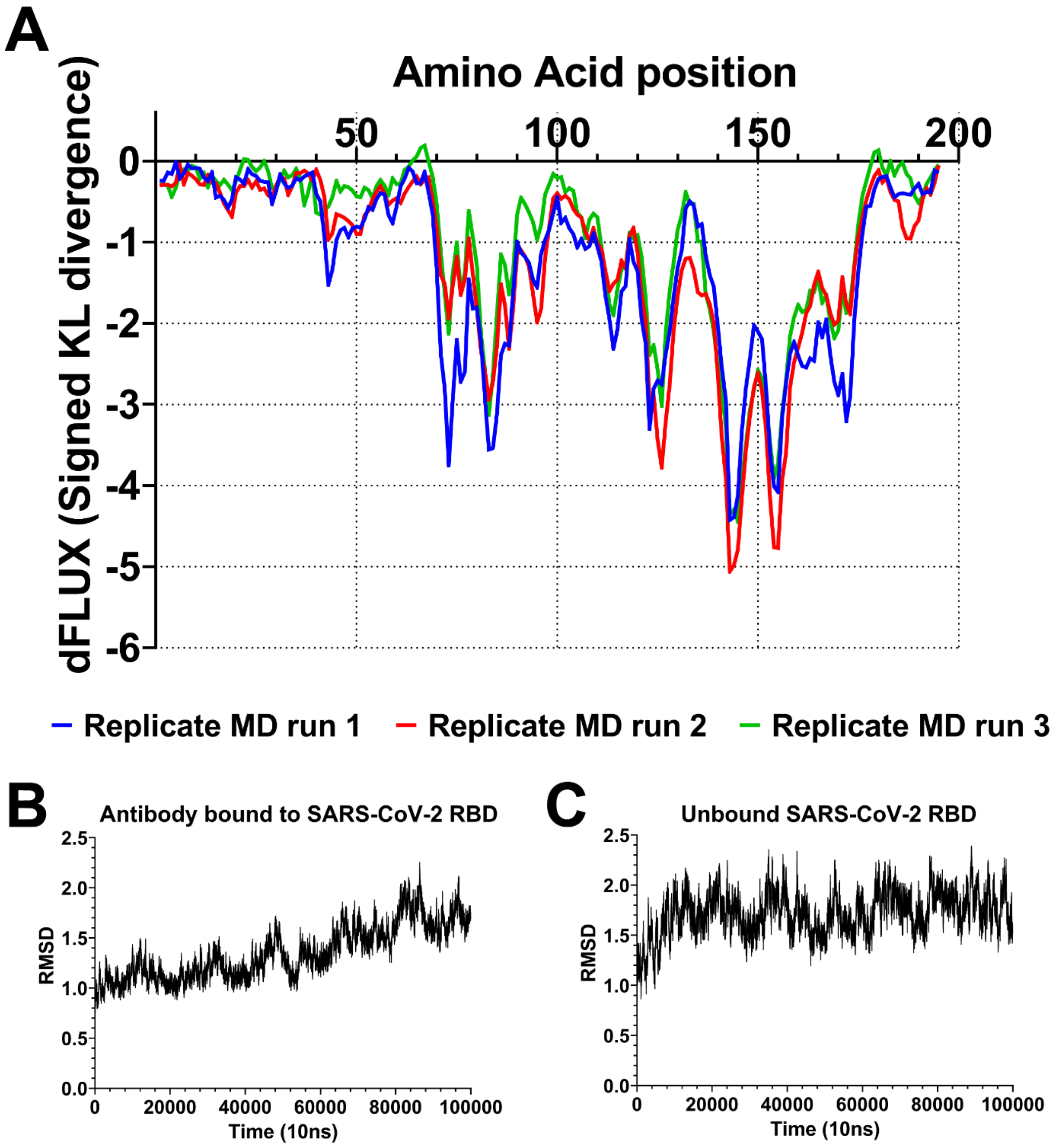

Supplemental Figure 4: Reproducibility/stability of the molecular dynamics (A) Three replicate binding signature profiles (i.e. negative KL divergence) demonstrating the reproducibility for identically initiated comparative molecular dynamics analysis of COVOX-222 bound SARS-CoV-2. Characteristic RMSD plots for the 10ns equilibration of the (B) antibody-bound and (C) unbound SARS-CoV-2 is also shown.
