# Supplementary material for: Identifying vaccine escape sites via statistical comparisons of short-term molecular dynamics
Source: Biophys Rep (N Y). 2022 Apr 4;2(2):100056. doi: 10.1016/j.bpr.2022.100056 (PMC8978532; doi:10.1016/j.bpr.2022.100056)
Supplement: Document S2. Article plus supporting material [file mmc3.pdf]

Biophysical Reports

ARTICLE

Identifying vaccine escape sites via statistical comparisons of short-term molecular dynamics

Madhusudan Rajendran,[1,*] Maureen C. Ferran,[1] and Gregory A. Babbitt[1,*]
[1]Thomas H. Gosnell School of Life Sciences, Rochester Institute of Technology, Rochester, New York

ABSTRACT The identification of viral mutations that confer escape from antibodies is crucial for understanding the interplay between immunity and viral evolution. We describe a molecular dynamics (MD)-based approach that goes beyond contact mapping, scales well to a desktop computer with a modern graphics processor, and enables the user to identify functional protein sites that are prone to vaccine escape in a viral antigen. We first implement our MD pipeline to employ site-wise calculation of Kullback-Leibler divergence in atom fluctuation over replicate sets of short-term MD production runs thus enabling a statistical comparison of the rapid motion of influenza hemagglutinin (HA) in both the presence and absence of three well-known neutralizing antibodies. Using this simple comparative method applied to motions of viral proteins, we successfully identified in silico all previously empirically confirmed sites of escape in influenza HA, predetermined via selection experiments and neutralization assays. Upon the validation of our computational approach, we then surveyed potential hotspot residues in the receptor binding domain of the SARS-CoV-2 virus in the presence of COVOX-222 and S2H97 antibodies. We identified many single sites in the antigen-antibody interface that are similarly prone to potential antibody escape and that match many of the known sites of mutations arising in the SARS-CoV-2 variants of concern. In the Omicron variant, we find only minimal adaptive evolutionary shifts in the functional binding profiles of both antibodies. In summary, we provide an inexpensive and accurate computational method to monitor hotspots of functional evolution in antibody binding footprints.

WHY IT MATTERS Critical to public health is understanding how the rapid evolution of viruses allows them to mutate and subsequently escape our vaccines. Recently, high-throughput "wet bench" mutational scanning has demonstrated that vaccine escape is mitigated mostly by changes to just a few single sites on viral proteins that have large consequential effects on antibody binding. While some complicated and potentially biased scoring and machine learning approaches have been proposed for finding sites that support the "lock and key" relation between functional antibodies and their viral protein targets, we introduce a novel approach that relies upon a simple statistical comparison between the computer simulated motions or "molecular dynamics" of the target protein in both its antibody-bound versus its unbound state.

INTRODUCTION

In current attempts to prevent the spread of the coronavirus disease 2019 (COVID-19) pandemic, caused by severe acute respiratory syndrome coronavirus 2 (SARS-CoV-2), clinicians and scientists have focused their efforts on the development of vaccines that are hoped to induce broad, long-lasting, neutralizing antibodies. However, the selective pressures imposed by the presence of the neutralizing antibodies in the host population can also drive the evolution of viruses toward adaptations that allow them to escape neutralization. For example, in the case of influenza viruses, immunity is provided by antibodies that target the hemagglutinin (HA), responsible for viral attachment and viral fusion to the host cell. However, these antibodies drive selection for amino acid substitutions in the HA, causing the influenza virus to rapidly evolve every year (1). Similarly, the antibody selection by the host immune system can also drive the emergence of new SARS-CoV-2 variants. Therefore, when developing vaccines that elicit antibodies against a broad range of strains, research efforts should also be aimed at identifying potential mutations that can facilitate viral escape from the neutralization effects of specific antibodies (2,3).

Submitted December 15, 2021, and accepted for publication March 31, 2022.

*Correspondence: madhusudan.rajendran@gmail.com or gabsbi@rit.edu

Editor: Hagen Hofmann.

https://doi.org/10.1016/j.bpr.2022.100056

© 2022 The Author(s).

This is an open access article under the CC BY license (http://creativecommons.org/licenses/by/4.0/).

The traditional approach to identifying these mutations is by passaging the virus in the presence of antibodies in a directed selection experiment, followed by validation of the variants that arise with neutralization assays. For example, in influenza viruses, escape mutant selection using a panel of monoclonal antibodies (mAbs) was used to identify the five major antigenic regions, Sa, Sb, Ca1, Ca2, and Cb (4–6). However, a significant drawback of this approach is that the directed selection typically only favors one of the many potential mutations that can escape a given antibody. Another approach is to test antibody binding to a panel of viral variants. In a typical 500-residue viral protein, there are about 10^4 potential single amino acid mutants (7). Creating all individual mutants and then testing the mutants against the antibodies is an impossible task, thus causing researchers to limit themselves to exploring only a small portion of protein space (e.g., examining only mutations to alanine) (8,9). Such studies cannot give a complete picture of the mutational spectra that can allow a virus to escape neutralization by a given antibody (10).

Functional evolutionary studies of viral vaccine escape are often supplemented with protein structural determination via x-ray crystallography or cryogenic electron microscopy and subsequent contact mapping of heavy atoms within a specific cutoff distance. While structural information regarding the details of antibody binding are often considered the gold standard in defining epitopes, particularly for patent application, it has also been understood that structure alone cannot completely define the individual sites or "hotspots" that drive interactions within given protein-protein interaction (PPI) binding interfaces (11). To better identify sites that control PPI, a host of computational methods have been developed. These include coevolutionary rate comparison to identify functional pairings of sites across the PPI interface (12), complex scoring methods to predict PPI specificity (13,14), and, more recently, graph network and machine learning (ML) methods aimed at easing this PPI scoring challenge (15,16). So, while structural biology can provide a static image to verify that an antibody physically contacts a viral protein, it often cannot provide complete information regarding which amino acid sites are more prone to single replacement mutations that may drive sudden evolutionary changes affecting vaccine efficacy (i.e., hotspot sites or residues). Although binding can be observed and verified in structure, a much richer picture of the functional sites of binding may be determined by the study of changes to their motion (i.e., molecular dynamics [MD]) upon the formation of the PPI interface.

We propose here that a supplementation of structural analysis with a proper comparative statistical study of a bound versus unbound protein's short-term dynamics may help to directly resolve these hotspots of PPI specificity in any given PPI interface without imparting the potential biases of scoring functions and/or ML training data sets. The prediction of hotspot residues is crucial as these sites on the antibody-antigen complex have a strong propensity to disrupt binding interactions within the antibody-antigen interface (17,18). Recently, it has been demonstrated, via site-directed selection experiments and neutralization assays, that these hotspot regions share a common biophysical feature. They all tend to harbor single amino acid sites that have significant large affects upon binding interactions in the antibody-antigen interface (19). Given this common feature of hotspot residues, we further hypothesize an important role for comparative MD simulations of the antibody-antigen interface to help predict potential vaccine escape mutations before they happen, allowing for important functional context to real-time sequence-based surveillance of current and future pandemics.

Here, we utilize a relatively simple method of comparative statistical analysis of MD simulations developed by our lab (DROIDS—detecting relative outlier impacts in dynamic simulation or DROIDS 4.0) that employs a site-wise Kullback-Leibler (KL) divergence metric and a multiple test-corrected two-sample Kolmogorov-Smirnov (KS) test to successfully validate previously known sites of antibody escape in the influenza HA (19–23). We then utilized our site-wise comparative MD approach to identify potential sites prone to antibody escape in the spike protein of SARS-CoV-2. Specifically, in the Omicron variant we were able to identify sites in the receptor binding domain (RBD) that support the binding efficiency to two general neutralizing antibodies and its competitive binding to the natural receptor, human angiotensin-converting enzyme 2 (hACE2). We also compare our dynamics-based method to a more traditional structure-based method of counting heavy atom contacts using distance cutoffs within the antibody-viral binding interface. In summary, we present a method to accurately identify hotspot residues that are prone to single point mutations with large functional effects upon the antibody-antigen binding interface and thus are likely preadapted to allow for vaccine escape. Identifying such residues in silico will be essential for prescreening the antigenic consequences of viral genetic variations and designing better vaccines that induce long-term and broadly neutralizing antibodies against viral pathogens.

METHODS

PDB structure, glycosylation, and model preparation

The protein structures used for the primary models for analyzing the MD of antibody interactions with HA and the SARS-CoV-2-RBD are listed in Fig. 1 *A* (24–29). Any crystallographic reflections were removed along with any other small molecules used in crystallization.

A

PDB ID	Antigen	Antibody	AA and position	-KL divergence	D value	p value
4GMS	A/Victoria/3/1975 H3 Head domain	S139/1	K156	-4.65	0.810	p < 0.001
			G158	-5.81	0.895	p < 0.001
			N193	-4.81	0.807	p < 0.001
3ZTJ	A/California/4/2009 H1 Stalk domain	FI6v3	E47	-2.41	0.647	p < 0.001
4HLZ	A/Japan/305/1957 H2 Stalk domain	C179	-	-	-	-
7OR9	SARS-CoV-2 RBD	COVOX-222	D405	-2.57	0.572	p < 0.001
			K417	-3.27	0.678	p < 0.001
			K458	-3.49	0.703	p < 0.001
			G476	-5.50	0.888	p < 0.001
			F486	-4.52	0.800	p < 0.001
			N501	-4.55	0.708	p < 0.001
7M7W	SARS-CoV-2 RBD	S2H97	N394	-4.25	0.760	p < 0.001
			D428	-3.81	0.692	p < 0.001
			K462	-3.37	0.723	p < 0.001
			E484	-5.20	0.837	p < 0.001
			L518	-6.56	0.945	p < 0.001
**PDB ID**	**Protein 1**	**Protein 2**	**AA and position**	**-KL divergence**	**D value**	**p value**
6VW1	SARS-CoV-2 RBD mutated to include VOC mutations	ACE2	K417N	-0.053	0.140	p < 0.001
			L452R	-0.206	0.155	p < 0.001
			T478K	-0.129	0.190	p < 0.001
			E484K	-0.204	0.165	p < 0.001
			N501Y	-0.208	0.130	p < 0.05

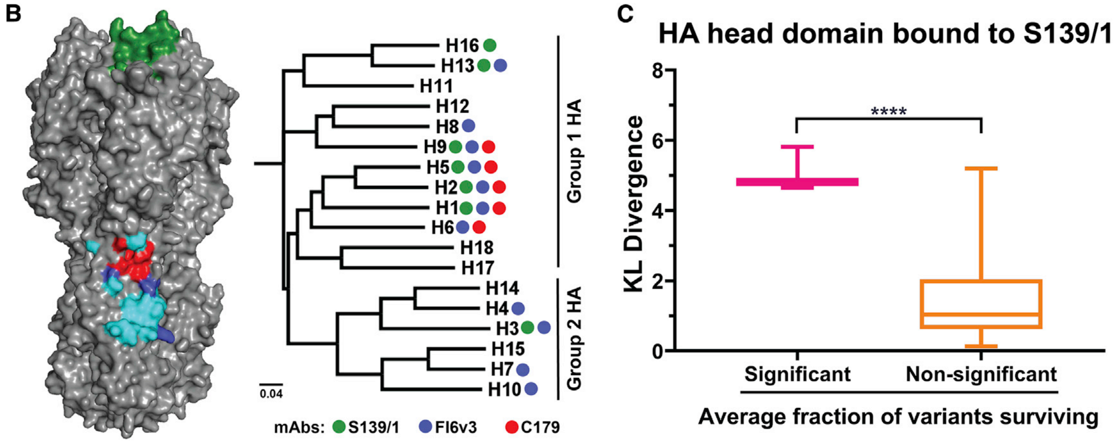

FIGURE 1 Description of PDB files and antibodies used in this study, and the comparison between KL divergence and average fraction of variants surviving.
(*A*) Table summarizing the protein structure used for primary models for analyzing the MD of antibody interactions with the influenza HA and SARS-CoV-2 RBD, and ACE2 interactions with wild-type and mutated SARS-CoV-2 RBD (includes VOC mutations). The table includes amino acid positions and the corresponding −KL divergence value denoting atomic fluctuations dampening for antibody-antigen/protein 1-protein 2 MD simulations. D value and the level of significance for the corresponding amino acid position are also given. (*B*) S139/1 (*green*), FI6v3 (*blue*), and C179 (*red*) epitopes are mapped onto the HA trimer, shown in gray (PDB: 1RVX). Overlapping epitopes between FI6v3 and C179 are shown in cyan. Next to the HA trimer is a phylogenetic tree of HA subtypes. Circles denote reported antibody binding or neutralization against that subtype. (*C*) Box and whisker plots showing the KL divergence values within 2 sd for amino acid sites that had significant average fraction of variants surviving and nonsignificant average fraction of variants in the presence of monoclonal antibody S139/1 (19). Only sites K156, G158, and N193 of the HA head domain had significant average fraction surviving viral particles.

Hydrogens were added, and crystallographic waters were removed using pdb4amber (AmberTools18) (30). Glycosylation was deleted using the swapaa function in UCSF Chimera 1.14 (31). Predicted glycosylation was rebuilt for the Amber force field using the glycoprotein builder on the glycam.org webserver and the GLYCAM06j-1 force field (32,33). Any missing loop structure in the files was inferred via homology modeling using the "refine loop" command to Modeler within UCSF Chimera (34,35). The globular head domain of the influenza HA is stable in its monomeric form. However, the stalk domain needs to be in a trimer to be stable (36). Therefore, MD simulations of PDB: 4GMS was performed using a trimmed (residues 57–270) monomeric form of the head domain. For MD simulations of the PDB: 3ZTJ and PDB: 4HLZ, the trimeric form of the stalk domain was used. The antibodies were trimmed, and only the heavy and light chains of the fragment antigen binding (Fab) were used. Finally, for the Omicron (B.1.1.529) variant simulations, we used the swapaa function to model the 15 RBD mutations onto PDB: 7OR9 (Fig. 4 *B*).

MD simulation protocols

All comparative MD analysis via our DROIDS pipeline was based upon 100 replicate sets of 1 ns accelerated MD runs (i.e., 100 × 1 ns MD runs in each comparative state, e.g., unbound versus bound). MD simulations were conducted using the particle mesh Ewald method via accelerated MD (pmemd.cuda) in Amber18 and the ff14SB protein

and GLYCAM06j-1 (32)) force fields and implemented on two RTX 2080 Ti graphics processor units controlled via a Linux Mint 19 operating system (32,37–41). These replicate sets were preceded by energy minimization, 300 ps of heating to 300 K, and 10 ns of equilibration, followed by random equilibration intervals for each replicate ranging from 0 to 0.5 ns. All protein systems were prepared via tLeAP (Ambertools18) and explicitly solvated, and charge neutralized with Na^+ and Cl^- ions in a Tip3P octahedral water box set to 12 nm beyond the surface of each protein with periodic boundaries (30,42). All simulations were regulated using the Anderson thermostat at 300 K and one atmospheric pressure (43). Root mean-square atom fluctuations and atom correlations were conducted in CPPTRAJ using the atomicfluct and atomicorr commands (44).

Comparative protein dynamics analyses with DROIDS 4.0 and statistical analyses

Comparative signatures of dampened atom fluctuation during antibody binding were presented as protein site-wise divergence in atom fluctuation in the antibody-bound versus unbound states for each viral target protein (both influenza HA and SARS-CoV-2 RBD). Divergences were calculated using the signed symmetric KL divergence calculation in DROIDS 4.0. This divergence or relative entropy (45) is taken between the homologous distributions of atom fluctuation (i.e., root mean-square fluctuation or rmsf taken from 0.01-ns time slices of total MD simulation time) comparing the MD of antibody-bound and unbound spike proteins averaged over the four protein backbone atoms, achieving individual amino acid resolution. The rmsf value is thus

$$rmsf = \frac{1}{4}\sum_{i=N,C,C_\alpha,O}^{4}\sqrt{\frac{1}{n}\sum_{j=1}^{n}\left(\left(v_{jx}-w_x\right)^2+\left(v_{jy}-w_y\right)^2+\left(v_{jz}-w_z\right)^2\right)}, \quad (1)$$

where v represents the set of XYZ atom coordinates for i backbone atoms (C, N, O, or Cα) for a given amino acid residue over j time points and w represents the average coordinate structure for each MD production run in a given ensemble. The KL divergence (i.e., relative entropy) or similarity between the rmsf of two homologous protein sites representing a functional binding interaction (i.e., where 0 = unbound state and 1 = antibody-bound state) can then be described by

$$KL_{divergence} = \sum_{t=10ps}^{T}\left[\left(rmsf_0 * \log\frac{rmsf_0}{rmsf_1}\right)+\left(rmsf_1 * \log\frac{rmsf_1}{rmsf_0}\right)\right]\Big/ T, \quad (2)$$

where rmsf represents the average root mean-square deviation of a given atom over time. More specifically, the rmsf is a directionless root mean-square fluctuation sampled over an ensemble of MD runs with similar time slice intervals. Since mutational events at the protein level are typically amino acid replacements, this calculation is useful if applied to resolution of single amino acids rather than single atoms. Note that amino acid side chain dynamics are ignored because only the backbone atoms (N, Cα, C, and O) are homologous between residues. However, because the sidechain atoms always attach to this backbone, rmsf still indirectly samples the dynamic effect of amino acid sidechain as they are still present in the simulation. The reference state of the protein is always unbound while the query state is bound. Therefore, the pairwise comparison represents the functional impact of binding on the unbound protein's normal motion in its solvated state. Thus, binding contact dampens the fluctuation of atoms at the sites of binding to the degree to which they are involved in the binding interaction. Multiple test-corrected two-sample KS tests are used to determine the statistical significance of local site-wise differences in the rmsf distributions. As the analysis is site-wise and a statistical hypothesis test is conducted for each site, the Benjamini-Hochberg method (46) was used to adjust p values for the false discovery rate generated by the higher probability of false positive results generated by the analysis of the multiple sites of a given protein structure. Therefore, all significance tests and p values for these site-wise differences were calculated in DROIDS 4.0 using two-sample KS tests with the Benjamini-Hochberg multiple test correction in DROIDS 4.0. Further mathematical details of DROIDS 4.0 site-wise comparative protein dynamics analysis were published previously by our group and can be found here (21,22). This code is available at our GitHub web landing: https://gbabbitt.github.io/DROIDS-4.0-comparative-protein-dynamics/, and is also available at our GitHub repository https://github.com/gbabbitt/DROIDS-4.0-comparative-protein-dynamics.

We also use this method to compare the mutational impacts on the bound SARS-CoV-2 RBD/angiotensin-converting enzyme 2 (ACE2) interface for all current variants of concern (VOC) mutations. Thus, rather than determining the effect of binding on the viral protein dynamics, we instead determine the effect of the mutations on the viral protein dynamics when it is bound to its human target receptor ACE2.

Epitope map identification

Previously published articles were surveyed to identify the epitope mapping of the HA and SARS-CoV-2 RBD antibodies (Table 1). In brief, the identified epitopes were highlighted on the amino acid back bone of the corresponding antigen. These studies utilized some combination of experimental methods for epitope mapping that included structural determination, site-directed mutagenesis, pepscan analysis, hydrogen deuterium exchange, and cross-linking coupled with mass spectrometry.

TABLE 1 HA and SARS-CoV-2 RBD antibodies and the source of the identified epitopes

Antibody	Antigen	References
S139/1	HA head	(26)
FI6v3	HA stalk	(25)
C179	HA stalk	(47)
COVOX-222	SARS-CoV-2 RBD	(48)
S2H97	SARS-CoV-2 RBD	(29)

To further assess the relationship between the structures of the interfaces in our models and the changes in their dynamics upon binding, we used the ContPro webserver (49) to compute the closest distances between all amino acid sites within 6 Å proximity across the PPI interface of both our Covox222-RBD and S2H97-RBD models. We then correlated these interface distances to the change in dynamics upon binding at each amino acid site (i.e., dFLUX or KL divergence).

Relation of dampened atom motion and the number of heavy atom contacts in the antibody-viral binding interface

The AmberTools20 "nativecontacts" function was utilized to extract both native and nonnative heavy atom contacts for the first frame (i.e., static structure) and the subsequent 100,000 frames from a 20-ns MD simulation on the antibody-bound structures of the SARS-CoV-2 RBD and the COVX222 and S2H97 antibodies (i.e., providing both a static view of the contacts in space as well as a dynamic view of the contacts over time). We subsequently analyzed the correlations of our atom dampening metric (i.e., KL divergence) to both the static heavy atom contacts and the fraction of contacts over time. All supporting files, data, and custom code are supplied in a folder labeled "S3 [heavy atom contact analyses]" in the Data S1 file (data_RajendranFerranBabbitt_2022). The commands we used to invoke nativecontacts in the AmberTools program are given in the README.txt file in this folder. Fig. S3 *A* shows an overview of the pipeline, including the use of our custom parsing scripts written in python and our correlation analyses written in R language.

RESULTS

Using our comparative MD analysis pipeline (DROIDS 4.0), we successfully identified and validated previously known antibody escape sites in the head and stalk domains of the influenza HA. We then expanded the scope of our study to identify potential mutational sites prone to antibody escape in the spike protein of SARS-CoV-2 RBD and its recent Omicron genetic variant.

Computational validation of antibody escape mutation hotspots in influenza HA head and stalk domains

We first conducted comparative site-wise analyses of various HA viral spike protein dynamics in both antibody-bound versus unbound states (Fig. 1 *A*). We then applied our approach to anti-HA antibodies with a wide range of breadth against different influenza strains. Negative-signed KL divergences indicated universal dampening of atom fluctuation at key epitope sites in the antibody-bound state. A two-sample KS test corrected for the number of sites analyzed confirmed the statistical significance of these binding effects (Fig. 1 *A*). S139/1 is a one such mAb known to neutralize both group 1 (H1, H2, H5, H9, and H13) and group 2 (H3) influenza viruses (Fig. 1 *B*) (50). Crystallization studies have revealed that the antibody targets highly conserved residues in the RBD of the head domain (26) (Fig. 1 *B*). We applied a Mann-Whitney U-test to compare our KL divergence values, generated using MD simulations, to amino acid residues with significant average fraction of variants surviving and nonsignificant average fraction of variants surviving in the presence of S139/1, in accordance with (19) (Fig. 1 *C*). In our comparative analyses of MD simulations, we found significantly higher KL divergence in the average fraction of variants surviving during directed selection ($p < 0.001$), supporting that our KL divergence metric proves to be a useful quantitative measure in discriminating sites with mutations leading to vaccine escape. Comparative MD simulations of HA bound to S139/1 and unbound have revealed strong dampening of atom fluctuations occurring at sites K156, G158, and N193 of the HA, with $-$KL divergence values -4.65 ($D = 0.810, p < 0.001$), -5.81 ($D = 0.895, p < 0.001$), and -4.81 ($D = 0.807, p < 0.001$), respectively (Figs. 1 *A* and 2 *A*). Directed selection using mAb S139/1 revealed that these three residues match empirically identified sites of strong escape (Fig. 2 *B*) (19). As expected, the three amino acid residues with the highest negative KL divergence in our comparison of atom fluctuations from MD simulations also fall directly in the empirically determined physical binding footprint of the antibody. And they are the same three sites where previous works have selected escape mutants in the H1, H2, and H3 HAs (26,50). It should be noted that residue 156 is a part of the 150 loop of the influenza HA and forms electrostatic interactions by inserting itself into the acidic pocket in the Fab formed by residues Glu^{H35} and Glu^{H50}. Another 150 loop binding determinant is residue 158, which has the largest dampening of atomic fluctuations. Gly158 is closely stacked on the light chain complementarity determining region 3 of S139/1 and is further stabilized by a main chain hydrogen bond between S159 and Asn^{L92}. Thus, any mutations at these positions will cause clashes at the antibody interface. S139/1 also recognizes members of the 190 helix. In the residues of 190 helix, Asn193 plays a significant role in antibody recognition and is buried by heavy chain complementarity determining regions 1–3 of S139/1 (50). Furthermore, it should be noted that site 193 was known to

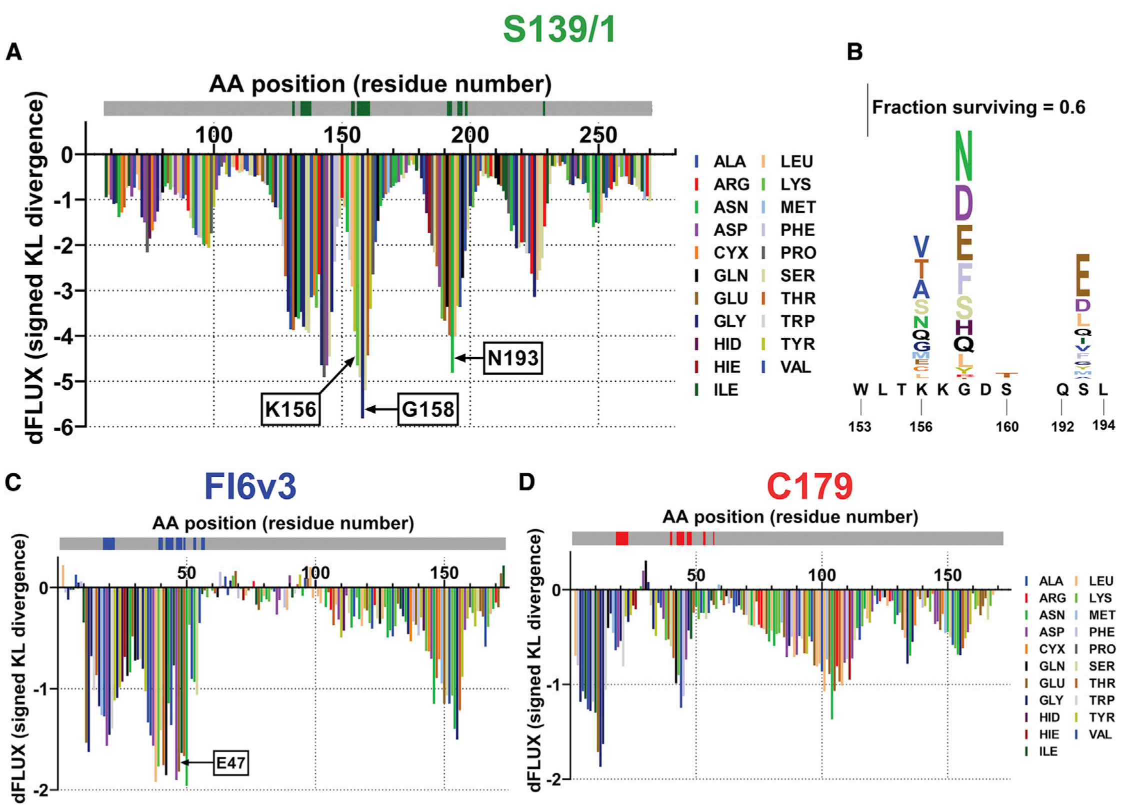

FIGURE 2 Epitope prediction and validation of hotspot residues of the influenza hemagglutinin in the presence of neutralizing antibodies. Sequence positional plotting of dampening of atom motion on the influenza hemagglutinin (HA) head domain by (*A*) S139/1 and (*B*) matching hotspots identified in lab work by Doud et al. (19) and additional sites of moderated dampening of atom motion on the HA stalk domain by (*C*) FI6v3 and (*D*) C179. The sequence profile of the −KL divergence between S139/1 and the head domain produces strong negative peaks in (*A*) at K156, G158, and N193. A modest negative peak is observed in the stalk domain in (*C*) at E47 in the presence of FI6v3. HA1 numbering is used to identify the amino acid positions in (*A*). HA2 numbering is used to identify the amino acid position in (*C*) and (*D*). The gray bar on top of the −KL divergence plots denotes the HA amino acid backbone with the location of (*A*) S139/1 epitopes shown in green, (*C*) FI6v3 epitopes in blue, and (*D*) C179 epitopes in red. (*B*) Logo plots of S139/1 show the amino acid position that has the largest effect. Letter heights are proportional to the excess fraction of virions with that mutation that survive the antibody, as indicated by scale bars. The logo plot was prepared using deep mutational scanning data from Doud et al. (19).

interact with the host receptor molecule (sialic acid moiety), suggesting the contribution of this residue to receptor binding of HA (51,52). Therefore, isolates with mutated residues at site 193 have reduced viral fitness due to their inability to bind to the host receptors (53). Selective pressure created by S139/1 on residue 193 has other secondary effects on the virus, such as the reduced binding activity of the influenza virus. With mAb S139/1, atomic fluctuation dampening is also seen in residues 133–134 of the influenza HA (Fig. 2 *A*). These residues are part of the 130 loop of the HA, and make a significant portion of the RBD of the influenza virus. Finally, in addition to atomic fluctuation dampening at residues 133–134 and at residues 156/158, we also see dampening at 145–147 (Fig. 2 *A*). Upon further examination, there lie no epitopes of S139/1 between residues 145 and 147, supporting that this dampening may be an artifact of the residue's location between two epitope sites (130 loop and 150 loop) of S139/1. Therefore, in summary, the three sites determined to have the largest physical effect upon antibody binding in our simulations (i.e., showing the largest negative KL divergence) are exactly the same three sites most likely to evolve antibody escape under directed selection experiments of (19).

We also chose two broad antibodies that target the stalk domain of the HA (FI6v3 and C179) to further validate our computational approach to identify potential sites of escape. FI6v3 was first isolated by high-throughput screening of immortalized antibody-secreting cells and was found to bind to both group 1 (H1, H2, H5, H6, H8, H9, and H13) and group 2 (H3, H4, H7, and H10) viruses (Fig. 1 *B*) (24). Antibody C179 was first isolated from a mouse that had been immunized with the H2N2 virus and was later found to cross-neutralize H1, H2, H5, H6, and H9 subtypes (Fig. 1 *B*) (47). Both FI6v3 and C179 have epitopes that lie in the stalk domain and are known to interfere with membrane fusion (Fig. 1 *B*). Our MD simulations do not reveal dampening of atomic fluctuations in the

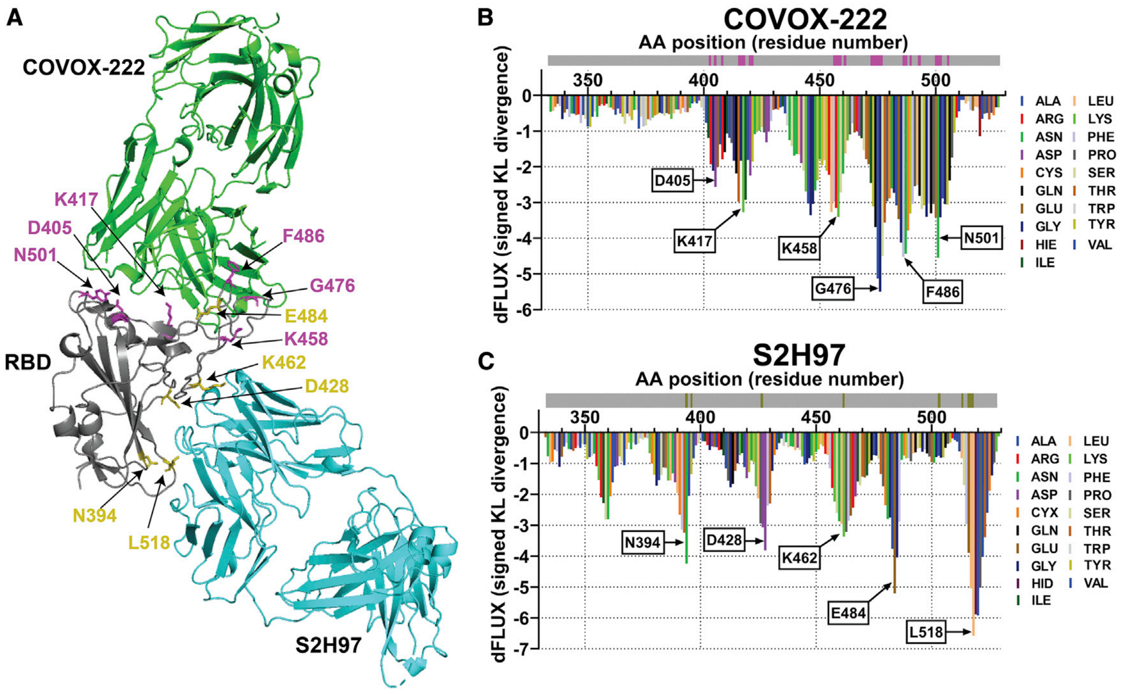

FIGURE 3 Identification of hotspot residues that escape neutralizing antibodies and the importance of the SARS-CoV-2 variants of concern mutations.
(*A*) Crystal structure of COVOX-222 (*green*) (PDB: 7OR9) and S2H97(*cyan*) (PDB: 7M7W) superimposed onto the structure of RBD of SARS-CoV-2 (*gray*) (PDB: 7M7W) (42,43). COVOX-222 epitopes with the greatest KL divergence dampening are highlighted in pink, and S2H97 epitopes with the greatest KL divergence dampening are highlighted in olive. Sequence positional plotting of dampening of atom motion on the RBD of SARS-CoV-2 by (*B*) COVOX-222 and (*C*) S2H97. Amino acid positions with moderate to modest dampening of atomic fluctuation are identified in (*B*) and (*C*). The gray bar on the top of the KL divergence plots denotes the RBD domain amino acid backbone with the location of COVOX-222 epitopes shown in pink and the S2H97 epitopes shown in olive.

stalk domain in the presence of FI6v3 or C179. When we look at the difference in atomic fluctuations of the stalk antibodies on the same scale as S139/1, we only see a few sites with only weak to moderate levels of dampening in atom fluctuation (Fig. 2, *C* and *D*). In conclusion, antibody selection experiments revealed very similar results as our comparative MD simulation studies, with only few sites in the stalk domain with slightly increased fraction of variants surviving mAb FI6v3 again, much in accordance with the experiments of (19). As a result, these authors concluded that the stalk domain is less capable of escaping antibodies by single mutations (19). We also find that, in the presence of FI6v3, both directed selection and MD simulation show a small bump at site 47 (KL $= -2.41$, D $=$ 0.647, $p < 0.001$), which is of importance (Figs. 1A and 2C), also in accordance with (19). Other mutational scanning studies have demonstrated that the introduction of E47R in the stalk domain has increased the resistance to FI6v3 (54). Like these studies, we also conclude that the HA stalk is more intolerant of mutations and, confirmed by us here, by a weaker signature of negative KL divergence at individual sites with more dampened atom fluctuations upon binding (Fig. 2, *C* and *D*). The presence of sites with only small divergences in atomic fluctuations in our comparative MD simulations on the HA stalk would seem to confirm that the antistalk antibodies studied here readily target sites with high mutational tolerance, as suggested by other site-directed experiments (55), or that the binding energetics at protein-protein interfaces can be asymmetrically distributed across all sites, thus preventing us from identifying the mutational tolerant sites (17,18).

Computational prediction of antibody escape mutation hotspots in SARS-CoV-2 RBD

Upon validation of our computation approach using influenza HA, we implemented our computational pipeline to identify potential hotspot residues in the RBD of the spike protein of SARS-CoV-2 in the presence of two recently published antibodies against the virus: COVOX-222 and S2H97 (Fig. 3 *A*). COVOX-222 is known to bind to different residues than S2H97 and is known to neutralize strains P.1 (gamma) from Brazil, B.1.351 (beta) from South Africa, and B.1.1.7 (alpha) from the UK (48). Termed “super-antibody,” S2H97, is known to bind with high affinity across all sarbecovirus clades and prophylactically protects hamsters from

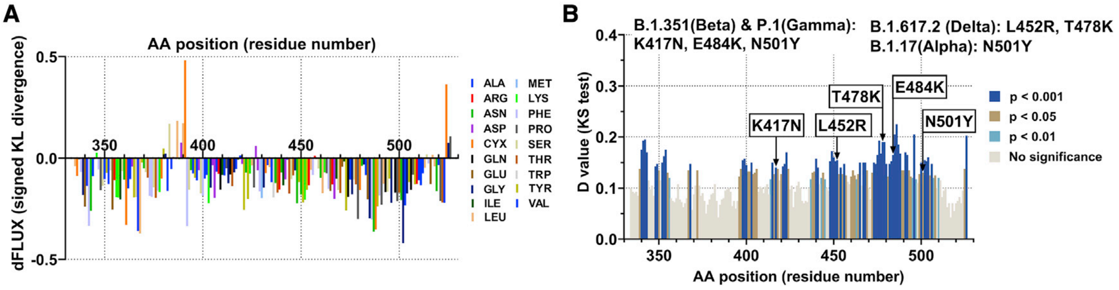

FIGURE 4 Functional impacts of SARS-CoV-2 VOC mutations on the MD of the SARS-CoV-2/ACE2 interface. (*A*) The site-wise KL divergence profiles showing the dampening of atom motion between the wild-type and mutated RBD of the spike protein of SARS-CoV-2 in the presence of hACE2. The wild-type RBD was computationally mutated to include mutations from the VOC. (*B*) Multiple test-corrected two-sample KS tests of significance for the impact of the mutations are also shown. Amino acid positions that correspond to the VOC mutations are highlighted and show higher level significance than other positions. Mutations that correspond to the four VOC in the RBD of spike protein are noted on top of (B).

viral challenge (29,56). In the presence of COVOX-222, we see the most dampening of atomic fluctuations at residues G476 (KL = −5.50, D = 0.888, $p < 0.001$), F486 (KL = −4.52, D = 0.800, $p < 0.001$), and N501 (KL = −4.55, D = 0.708, $p < 0.001$) of the RBD (Figs. 1 *A* and 3 *B*). We see modest amount of dampening at residues D405 (KL = −2.57, D = 0.572, $p < 0.001$), K417 (KL = −3.27, D = 0.678, $p < 0.001$), and K458 (KL = −3.49, D = 0.703, $p < 0.001$) of the RBD (Figs. 1 *A* and 3 *B*).

It should be noted that the residues picked up by our computational method are part of the epitopes of COVOX-222. Of all the residues identified by the comparison of MD simulation, the residues with the greatest dampening of atomic fluctuation have a higher chance of being classified as hotspot residues. They are more prone to mutate, thus allowing the virus to escape COVOX-222. Residue 417, one of the residues with moderate dampening of atomic fluctuation, makes a weak salt bridge interaction with the heavy chain complementarity determining region 3 residue E99 of COVOX-222 (48). Also, residue N501, one with residues with the most dampening of atomic fluctuations, is known to interact with light chain complementarity determining region 1 residue P30 of the antibody via a stacking interactions (48,57). In the case of the mAb S2H97, we see a moderate dampening of atomic fluctuations at sites D428 (KL = −3.81, D = 0.692, $p < 0.001$) and K462 (KL = −3.37, D = 0.723, $p < 0.001$). Sites N394 (KL = −4.25, D = 0.760, $p < 0.001$), E484 (KL = −5.20, D = 0.837, $p < 0.001$), and L518 (KL = −6.56, D = 0.945, $p < 0.001$) have the most dampening of atomic fluctuations (Figs. 1 *A* and 3 *C*). Four of the five sites, except E484, fall in the epitope footprint of S2H97 (29). Like mAb COVOX-222, we predict that the sites with the most dampening of atomic fluctuations (i.e., negative KL divergence) are more prone to functionally evolve under the selection pressure of the vaccine, thus allowing the virus to potentially escape the binding of S2H97. However, E484 does not fall in the S2H97 footprint; it has been shown that the mutation of this site is known to enhance immune escape form neutralizing antibodies and also increase affinity to hACE2 (58).

Many of the residues identified as potential sites of escape, either in the presence of COVOX-222 or S2H97, overlap with mutations seen in the VOC. At present, there are mainly five kinds of VOC: Alpha, Beta, Gamma, Delta, and Omicron. We employed comparative MD simulation of the SARS-CoV-2 RBD bound to hACE2 (PDB: 6VW1 trimmed to only include the viral RBD) modeled with and without the recently arising VOC mutations in the alpha to delta variant strains. Three of the VOC mutations (417, 484, and 501) that were previously identified as potential escape sites using our approach (Figs. 1 *A*, 3, *B* and *C*) here were shown to significantly increase viral binding to hACE2 at the protein interface. This additional comparative dynamics analysis of wild-type versus mutant viral bound structures demonstrates that the VOC mutations have an overall effect to increase binding at the protein interface (i.e., overall negative KL divergence in Fig. 4 *A*). In addition to the three sites that we confirmed previously by comparing bound versus unbound dynamics (i.e., 417, 484, and 501), we also saw significant differences in the viral bound atomic fluctuations (i.e., impacts of mutations themselves) at positions 452 (KL = −0.206, D = 0.155, $p < 0.001$) and 478 (KL = −0.129, D = 0.190, $p < 0.001$) of RBD (Fig. 4 *B*). Investigation with pseudoviruses possessing RBD mutations harbored by VOC demonstrated that the plasma-neutralizing activity of vaccinated individuals showed one- to threefold significant decreases against E484K, N501Y, or the K417N + E484K + N501Y triple mutant (59). These results confirm what we have observed using our comparative MD analysis (Figs. 1 *A* and 3, *A–D*) and extend our method to the investigation of biophysical impacts on function the individual VOC mutations

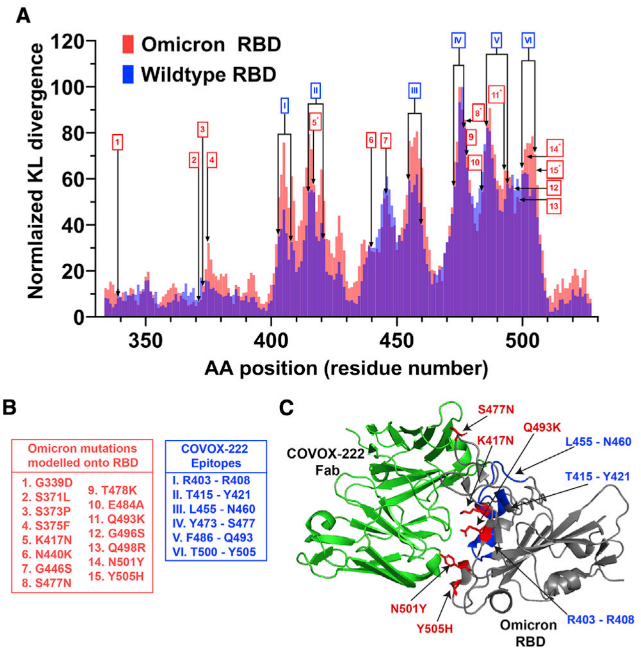

FIGURE 5 MD simulations with the Omicron variant reveal sites that promote similar binding affinity to hACE2.
(*A*) Sequence positional plotting of the normalized dampening of atom motions on the Omicron RBD (*red*) and the wild-type RBD (*blue*) by monoclonal antibody COVOX-222. KL divergence values for normalization of the wild-type RBD were obtained from Fig. 3 *B*, and for the Omicron RBD they were obtained from Fig. S1 *A*. The Omicron RBD mutations are labeled in red (1–15). The sites corresponding to the COVOX-222 epitope on the wild-type RBD on labeled in blue (I–VII). The amino acid residues that correspond to the numbers and roman numerals are listed in (*B*). In the Omicron RBD, we see an increase in atomic fluctuation peaks at several of the epitope sites. Sites that overlap between the Omicron mutations and COVOX-222 epitope (shown by *) are sites of ACE2 interaction. (*B*) List of Omicron mutations modeled onto RBD, and the list of COVOX-222 epitopes. (*C*) Crystal structure of COVOX-222 Fab (*green*) superimposed onto the structure of the Omicron RBD (*gray*) (PDB: 7NXA). Some of the amino acid residues that correspond to the COVOX-222 epitopes, which show an increase in atomic fluctuation dampening, are shown in blue. Several of the amino acid residues that correspond to Omicron mutations with increased atomic fluctuation dampening, which are sites of ACE2 interactions, are shown in red.

themselves (Fig. 4, *A* and *B*). Furthermore, evidence from clinical trials on the impact of VOC on vaccine efficacy confirms what we have observed as well. For example, the ChAdOx1 nCoV-19 vaccine and the single-dose JNJ-78436735 (Johnson & Johnson/Janssen) vaccine have reduced vaccine efficacy by 10.4 and 57%, respectively, against the B.1.351 variant, which contains K417N, E484K, and N501Y mutations (60–62). Therefore, when evaluating vaccine efficacy and the effect of neutralizing antibodies against the SARS-CoV-2 virus, the focus should be given to amino acid positions that are prone to mutate and escape the antibody, as these positions might cause the emergence of new VOCs.

Finally, we also modeled the most recent VOC, Omicron. The Omicron RBD is known to harbor 15 different mutations, with several mutations linked to greater transmissibility, lower vaccine efficiency, and increased risk of reinfection (63). To further understand the evolution of the variant, we ran comparative MD simulations with the Omicron variant bound and unbound to mAb COVOX-222. In the wild-type RBD we see a dampening of atomic fluctuation at residues that correspond very well to the antibody footprint (Fig. 5 *A* and *B*). Interestingly, in the Omicron RBD, we see some increase in the atomic fluctuation dampening at several of the residues that correspond to the COVOX-222 epitopes (Fig. 5). Furthermore, some of the mutations that correspond to the Omicron RBD overlap with the COVOX-222 antibody footprint. These sites include K417N ($KL = -2.60$, $D = 0.631$, $p < 0.001$), S477N ($KL = -3.83$, $D = 0.762$, $p < 0.001$), Q493K ($KL = -2.84$, $D = 0.762$, $p < 0.001$), N501Y ($KL = -3.25$, $D = 0.737$, $p < 0.001$), and Y505H ($KL = -3.16$, $D = 0.77$, $p < 0.001$) (Figs. 5 and S1). Upon closer examination, the residues that overlap between the antibody footprint and the Omicron mutations are sites where the hACE2 is known to interact with the RBD. As a result, the increase in atomic fluctuation dampening might not be because of the direct evolution of stronger binding to the antibody, but also due to the evolution of increased binding affinity to hACE2. Other studies have shown that the mutations in the Omicron variant increase the number of salt bridges and hydrophobic interactions between RBD and hACE2, resulting in a higher binding efficiency to hACE2 (64). In addition, it has also been shown that the structural changes in the RBD domain, caused by the mutations, reduce the antibody interactions (65,66). We present a similar analysis of the functional binding of the Omicron variant with S2H97 (Fig. S2), which also indicates minimal overall effects of the genetic changes on potential antibody neutralization, with the exception of E484A, T478K, and S477N sites, in which Omicron appears to have lost interaction with S2H97.

To provide comparison of our results with more traditional structure-based methods of contact mapping and counting heavy atom contacts within the structure of the binding interface, we conducted native and nonnative heavy atom contact analysis using AmberTools20. We report moderate and significant correlations between the degree of dampened atom motion (KL divergence) and the number of static heavy atom contacts, indicating roughly 25% association (Table S1 and Fig. S3 *B*). We do not find that counting the fraction of static contacts over time of 20 ns adds any more information, probably not surprising because the bound protein structures are well equilibrated and do not largely change in structural conformation over time (Fig. S3 *B*). However, we do find that both the

number of sites identified, as well as the slopes of the linear models describing the relation between heavy atom counts and KL divergence are quite dependent upon the distance cutoff chosen for counting (Fig. S3, *B*, *C*, and *D*, respectively) While some sites identified by both methods are quite congruent (Fig. S3, *C* and *D*) when a good cutoff distance is chosen, we conclude that our analyses of short-term dynamics is better than contact mapping in its ability to fully identify the larger landscape of sites that may be prone to vaccine escape. Our method is also not prone to potential bias imparted by the user's arbitrary choice of cutoff distances in defining heavy atom contacts. Finally, we also demonstrate that our results are quite reproducible over identically initiated sets of replicate MD runs (Fig. S4). In summary, we observe that introduction of mutations sites in the Omicron RBD have only slightly altered the COVOX-222 and S2H97 interactions with the viral RBD. Most importantly, our computational method appears to be an important, reproducible, and effective way to prescreen, quantify and monitor these changes without much additional effort in the lab.

DISCUSSION

Here, we report a remarkably fine agreement between experimental approaches to identify vaccine escape mutations and a relatively simple comparative computation applied to MD simulations conducted on the primary functional structural states of antibody-antigen interfaces. Furthermore, we demonstrate the capacity of comparative MD to describe the overall trajectory of evolution of the SARS-CoV-2/ACE2 binding interface throughout the ongoing pandemic. While some of what we report might be able to be deduced from structural data alone, our additional comparative analysis of the dynamic states induced by the structure-function relationship adds a clear and quantitatively grounded level of resolution allowing for the identification of single sites (i.e., hotspots) contributing to the epitope binding of antibodies. Directed selection experiments, followed by confirmation through neutralization assays, are the classical approach for identifying the location of these hotspot residues. Not only are the selection experiments time consuming, unless they are conducted in a sophisticated high-throughput manner, they only identify one of many potential mutations that may escape an antibody (67). Compared with the classical method, our simple computational approach can identify the locations of sites prone to vaccine escape in a matter of days on modern GPU hardware (e.g., 30 h on an Nvidia RTX 3080Ti). In addition, our method identifies positions of all amino acids that could mutate to escape the antibody in a single in silico experiment. And, compared with other computational tools, our MD-based approach does not require large and potentially biased ML training data sets. Furthermore, the de novo prediction is based on a given experimental structure, which enables an unprecedented close synergy between our computational approach and existing laboratory methods for identifying potential routes of viral evolution leading to enhanced transmissibility and vaccine escape.

Over the years, many sequence- and structure-based computational methods have been developed for the purpose of deducing protein sites contributing to PPI specificity. These have ranged from coevolutionary rate analysis (12), simple contact mapping combined with a variety of contact scoring methods (13,68,69), and, more recently, graph network and ML approaches aimed at solving the "contact scoring challenge" (15,16) originally raised by the work of Bogan and Thorn (11). In addition, many tools that have been more specifically designed for epitope prediction also include sequence-based methods, ML methods, and structure-based methods. The epitope surface accessibility to antibody binding is generally used by the sequence-based methods (70,71). The availability of an antigen sequence is crucial for the sequence-based method; however, the predicted epitope residues are not grouped into the corresponding epitopes (72). The ML-based epitope prediction methods include several steps: a collection of data sets with clean and comprehensive data, extraction of antigen features of the sequence (e.g., physicochemical properties, evolutionary information, amino acid composition), and training the model using ML algorithms (73). Some of the commonly used ML tools for epitope prediction are ABCPred (uses artificial neural network), COBEpro (uses support vector machine), EPSVR (uses support vector regression method), and BepiPred (based on random forest algorithm) (70,71,74–76). Finally, structure-based methods identify epitopes by antigen structure and epitope-related propensity scales, including specific physicochemical properties and geometric attributes (71,77). As the evolution of antibody-antigen interactions seems largely driven by single-site mutations with large functional antibody-antigen binding effects that would impart obvious biophysical impacts on MD, we offer a simple and effective alternative computational method to study vaccine escape in silico using readily available MD simulation software. We have combined this with a classic and easily interpretable approach to the statistical comparison of distributions of local atom fluctuations on virus proteins in the presence/absence of antibodies. While our comparative dynamics method may be computationally heavy compared with many of these, it is not burdened by potential bias imparted by the data sets used to develop the contact scoring method or to train ML algorithms. However, it is subject to the main caveats of all MD studies, mainly that the force fields

employed are accurate for the systems being modeled, and that the dynamics of the systems are appropriately stabilized and representatively sampled.

In summary, we have computationally identified hotspot residues known to mutate in a viral antigen in the presence of a neutralizing antibody. We first validated our approach using the influenza spike protein and three well-characterized antibodies against the influenza HA (13). We then implemented our approach to identify sites in the RBD of SARS-CoV-2 that are known to mutate in the presence of neutralizing mAbs S2H97 and COVOX-222 (42,43). We further identified that residues known to mutate in the presence of the antibody overlap with the mutations seen in the VOC. Finally, we also identified sites in the Omicron variant mutations that enhance binding efficiency to hACE2. While the method described here is not a complete substitute for laboratory-based methods and takes longer to conduct than simple analyses of antibody-bound viral protein structure, we believe it can greatly complement these methods by allowing time and cost saving through the computational prescreening of the underlying biophysics that may drive outcomes of many potential lab experiments. Determining which viral mutations escape from antibodies will be crucial for designing future therapeutics and vaccines and assessing future antigenic implications of ongoing viral evolution.

SUPPORTING MATERIAL

Supporting material can be found online at https://doi.org/10.1016/j.bpr.2022.100056.

AUTHOR CONTRIBUTIONS

M.R. wrote the manuscript, designed and performed the experiments, and analyzed the data. G.A.B. developed the code, revised and edited the manuscript, designed the experiments, and analyzed the data. M.C.F. edited the manuscript and analyzed the data.

DECLARATION OF INTERESTS

The authors declare no competing interests.

ACKNOWLEDGMENTS

We acknowledge Dr. Ernest Fokoue (School of Mathematical Sciences, Rochester Institute of Technology) for his consultation on the prior development of our method. We also acknowledge Dr. Feng Cui and Dr. André O. Hudson (Thomas H. Gosnell School of Life Sciences, Rochester Institute of Technology) for helpful comments regarding this work. There are no funding sources for this work. The authors also acknowledge Research Computing at the Rochester Institute of Technology for providing computational resources and support

REFERENCES

1. Smith, D. J., A. S. Lapedes, …, R. A. M. Fouchier. 2004. Mapping the antigenic and genetic evolution of influenza virus. *Science.* 305:371–376.
2. Krammer, F., and P. Palese. 2015. Advances in the development of influenza virus vaccines. *Nat. Rev. Drug Discov.* 14:167–182.
3. Łuksza, M., and M. Lässig. 2014. A predictive fitness model for influenza. *Nature.* 507:57–61.
4. Caton, A. J., G. G. Brownlee, …, W. Gerhard. 1982. The antigenic structure of the influenza virus A/PR/8/34 hemagglutinin (H1 subtype). *Cell.* 31:417–427.
5. Gerhard, W., J. Yewdell, …, R. Webster. 1981. Antigenic structure of influenza virus haemagglutinin defined by hybridoma antibodies. *Nature.* 290:713–717.
6. Webster, R. G., and W. G. Laver. 1980. Determination of the number of nonoverlapping antigenic areas on Hong Kong (H3N2) influenza virus hemagglutinin with monoclonal antibodies and the selection of variants with potential epidemiological significance. *Virology.* 104:139–148.
7. Haddox, H. K., A. S. Dingens, and J. D. Bloom. 2016. Experimental Estimation of the Effects of All Amino-Acid Mutations to HIV's Envelope Protein on Viral Replication in Cell Culture. *PLoS Pathog.* 12:e1006114.
8. Pierce, B. G., Z.-Y. Keck, …, S. K. H. Foung. 2016. Global mapping of antibody recognition of the hepatitis C virus E2 glycoprotein: Implications for vaccine design. *Proc. Natl. Acad. Sci. U. S. A.* 113:E6946–E6954.
9. Walker, L. M., S. K. Phogat, …, D. R. Burton. 2009. Broad and potent neutralizing antibodies from an African donor reveal a new HIV-1 vaccine target. *Science.* 326:285–289.
10. Polonis, V. R., B. K. Brown, …, N. L. Michael. 2008. Recent advances in the characterization of HIV-1 neutralization assays for standardized evaluation of the antibody response to infection and vaccination. *Virology.* 375:315–320.
11. Bogan, A. A., and K. S. Thorn. 1998. Anatomy of hot spots in protein interfaces. *J. Mol. Biol.* 280:1–9.
12. Ovchinnikov, S., H. Kamisetty, and D. Baker. 2014. Robust and accurate prediction of residue–residue interactions across protein interfaces using evolutionary information. *eLife.* 3:e02030.
13. Dhawanjewar, A. S., A. A. Roy, and M. S. Madhusudhan. 2020. A knowledge-based scoring function to assess quaternary associations of proteins. *Bioinformatics.* 36:3739–3748.
14. Nadalin, F., and A. Carbone. 2018. Protein-protein interaction specificity is captured by contact preferences and interface composition. *Bioinforma. Oxf. Engl.* 34:459–468.
15. Cao, Y., and Y. Shen. 2020. Energy-based graph convolutional networks for scoring protein docking models. *Proteins.* 88:1091–1099.
16. Geng, C., Y. Jung, …, L. C. Xue. 2020. iScore: a novel graph kernel-based function for scoring protein-protein docking models. *Bioinforma. Oxf. Engl.* 36:112–121.
17. Dall'Acqua, W., E. R. Goldman, …, R. A. Mariuzza. 1998. A mutational analysis of binding interactions in an antigen-antibody protein-protein complex. *Biochemistry.* 37:7981–7991.
18. Jin, L., B. M. Fendly, and J. A. Wells. 1992. High resolution functional analysis of antibody-antigen interactions. *J. Mol. Biol.* 226:851–865.
19. Doud, M. B., J. M. Lee, and J. D. Bloom. 2018. How single mutations affect viral escape from broad and narrow antibodies to H1 influenza hemagglutinin. *Nat. Commun.* 9:1386.
20. Babbitt, G. A., M. L. Lynch, …, A. O. Hudson. 2022. Function and evolution of B-Raf loop dynamics relevant to cancer recurrence under drug inhibition. *J. Biomol. Struct. Dyn.* 40:468–483.

21. Babbitt, G. A., E. P. Fokoue, …, L. E. Adams. 2020. DROIDS 3.0—Detecting Genetic and Drug Class Variant Impact on Conserved Protein Binding Dynamics. *Biophys. J.* 118:541–551.

22. Babbitt, G. A., J. S. Mortensen, …, J. K. Liao. 2018. DROIDS 1.20: A GUI-Based Pipeline for GPU-Accelerated Comparative Protein Dynamics. *Biophys. J.* 114:1009–1017.

23. Rynkiewicz, P., M. L. Lynch, …, G. A. Babbitt. 2021. Functional binding dynamics relevant to the evolution of zoonotic spillovers in endemic and emergent Betacoronavirus strains. *J. Biomol. Struct. Dyn.* 1–19. https://doi.org/10.1080/07391102.2021.1953604.

24. Corti, D., J. Voss, …, A. Lanzavecchia. 2011. A neutralizing antibody selected from plasma cells that binds to group 1 and group 2 influenza A hemagglutinins. *Science.* 333:850–856.

25. Dreyfus, C., D. C. Ekiert, and I. A. Wilson. 2013. Structure of a Classical Broadly Neutralizing Stem Antibody in Complex with a Pandemic H2 Influenza Virus Hemagglutinin. *J. Virol.* 87:7149–7154.

26. Lee, P. S., R. Yoshida, …, I. A. Wilson. 2012. Heterosubtypic antibody recognition of the influenza virus hemagglutinin receptor binding site enhanced by avidity. *Proc. Natl. Acad. Sci. U. S. A.* 109:17040–17045.

27. Liu, C., H. M. Ginn, …, G. R. Screaton. 2021. Reduced neutralization of SARS-CoV-2 B.1.617 by vaccine and convalescent serum. *Cell.* 184:4220–4236.e13.

28. Shang, J., G. Ye, …, F. Li. 2020. Structural basis of receptor recognition by SARS-CoV-2. *Nature.* 581:221–224.

29. Starr, T. N., N. Czudnochowski, …, G. Snell. 2021. SARS-CoV-2 RBD antibodies that maximize breadth and resistance to escape. *Nature.* 597:97–102.

30. Case, D. A., T. E. Cheatham, …, R. J. Woods. 2005. The Amber biomolecular simulation programs. *J. Comput. Chem.* 26:1668–1688.

31. Pettersen, E. F., T. D. Goddard, …, T. E. Ferrin. 2004. UCSF Chimera–a visualization system for exploratory research and analysis. *J. Comput. Chem.* 25:1605–1612.

32. Kirschner, K. N., A. B. Yongye, …, R. J. Woods. 2008. GLYCAM06: a generalizable biomolecular force field. Carbohydrates. *J. Comput. Chem.* 29:622–655.

33. Woods Group. 2005. GLYCAM Web. Complex Carbohydrate Research Center, University of Georgia, Athens, GA.

34. Fiser, A., R. K. Do, and A. Sali. 2000. Modeling of loops in protein structures. *Protein Sci. Publ. Protein Soc.* 9:1753–1773.

35. Sali, A., and T. L. Blundell. 1993. Comparative protein modelling by satisfaction of spatial restraints. *J. Mol. Biol.* 234:779–815.

36. Aartse, A., D. Eggink, …, M. J. van Gils. 2021. Influenza A Virus Hemagglutinin Trimer, Head and Stem Proteins Identify and Quantify Different Hemagglutinin-Specific B Cell Subsets in Humans. *Vaccines.* 9:717.

37. Darden, T., D. York, and L. Pedersen. 1993. Particle mesh Ewald: An N•log(N) method for Ewald sums in large systems. *J. Chem. Phys.* 98:10089–10092.

38. Götz, A. W., M. J. Williamson, …, R. C. Walker. 2012. Routine Microsecond Molecular Dynamics Simulations with AMBER on GPUs. 1. Generalized Born. *J. Chem. Theor. Comput.* 8:1542–1555.

39. Maier, J. A., C. Martinez, …, C. Simmerling. 2015. ff14SB: Improving the Accuracy of Protein Side Chain and Backbone Parameters from ff99SB. *J. Chem. Theor. Comput.* 11:3696–3713.

40. Pierce, L. C. T., R. Salomon-Ferrer, …, R. C. Walker. 2012. Routine Access to Millisecond Time Scale Events with Accelerated Molecular Dynamics. *J. Chem. Theor. Comput.* 8:2997–3002.

41. Salomon-Ferrer, R., A. W. Götz, …, R. C. Walker. 2013. Routine Microsecond Molecular Dynamics Simulations with AMBER on GPUs. 2. Explicit Solvent Particle Mesh Ewald. *J. Chem. Theor. Comput.* 9:3878–3888.

42. Wang, J., W. Wang, …, D. A. Case. 2006. Automatic atom type and bond type perception in molecular mechanical calculations. *J. Mol. Graph. Model.* 25:247–260.

43. Andersen, H. C. 1980. Molecular dynamics simulations at constant pressure and/or temperature. *J. Chem. Phys.* 72:2384–2393.

44. Roe, D. R., and T. E. Cheatham. 2013. PTRAJ and CPPTRAJ: Software for Processing and Analysis of Molecular Dynamics Trajectory Data. *J. Chem. Theor. Comput.* 9:3084–3095.

45. Kullback, S., and R. A. Leibler. 1951. On Information and Sufficiency. *Ann. Math. Stat.* 22:79–86.

46. Benjamini, Y., and Y. Hochberg. 1995. Controlling the False Discovery Rate: A Practical and Powerful Approach to Multiple Testing. *J. R. Stat. Soc. Ser. B Methodol.* 57:289–300.

47. Smirnov, Y. A., A. S. Lipatov, …, E. C. Claas. 1999. An epitope shared by the hemagglutinins of H1, H2, H5, and H6 subtypes of influenza A virus. *Acta Virol.* 43:237–244.

48. Dejnirattisai, W., D. Zhou, …, G. R. Screaton. 2021. Antibody evasion by the P.1 strain of SARS-CoV-2. *Cell.* 184:2939–2954.e9.

49. Firoz, A., A. Malik, …, V. Jha. 2010. ContPro: A web tool for calculating amino acid contact distances in protein from 3D -structures at different distance threshold. *Bioinformation.* 5:55–57.

50. Yoshida, R., M. Igarashi, …, A. Takada. 2009. Cross-Protective Potential of a Novel Monoclonal Antibody Directed against Antigenic Site B of the Hemagglutinin of Influenza A Viruses. *PLoS Pathog.* 5:e1000350.

51. Eisen, M. B., S. Sabesan, …, D. C. Wiley. 1997. Binding of the influenza A virus to cell-surface receptors: structures of five hemagglutinin-sialyloligosaccharide complexes determined by X-ray crystallography. *Virology.* 232:19–31.

52. Fleury, D., B. Barrère, …, M. Knossow. 1999. A complex of influenza hemagglutinin with a neutralizing antibody that binds outside the virus receptor binding site. *Nat. Struct. Biol.* 6:530–534.

53. Matrosovich, M., A. Tuzikov, …, Y. Kawaoka. 2000. Early alterations of the receptor-binding properties of H1, H2, and H3 avian influenza virus hemagglutinins after their introduction into mammals. *J. Virol.* 74:8502–8512.

54. Roubidoux, E. K., J. M. Carreño, …, F. Krammer. 2021. Mutations in the Hemagglutinin Stalk Domain Do Not Permit Escape from a Protective, Stalk-Based Vaccine-Induced Immune Response in the Mouse Model. *mBio.* 12:e03617–e03620.

55. Heaton, N. S., D. Sachs, …, P. Palese. 2013. Genome-wide mutagenesis of influenza virus reveals unique plasticity of the hemagglutinin and NS1 proteins. *Proc. Natl. Acad. Sci. U. S. A.* 110:20248–20253.

56. Kwon, D. 2021. This `super antibody' for COVID fights off multiple coronaviruses. *Nature* https://doi.org/10.1038/d41586-021-01917-9.

57. Shrestha, L. B., N. Tedla, and R. A. Bull. 2021. Broadly-Neutralizing Antibodies Against Emerging SARS-CoV-2 Variants. *Front. Immunol.* 12:752003.

58. Barton, M. I., S. A. MacGowan, …, P. A. van der Merwe. 2021. Effects of common mutations in the SARS-CoV-2 Spike RBD and its ligand, the human ACE2 receptor on binding affinity and kinetics. *eLife.* 10:e70658.

59. Wang, Z., F. Schmidt, …, M. C. Nussenzweig. 2021. mRNA vaccine-elicited antibodies to SARS-CoV-2 and circulating variants. *Nature.* 592:616–622.

60. Emary, K. R. W., T. Golubchik, …, A. J. Pollard. 2021. Efficacy of ChAdOx1 nCoV-19 (AZD1222) vaccine against SARS-CoV-2 variant of concern 202012/01 (B.1.1.7): an exploratory analysis of a randomised controlled trial. *The Lancet.* 397:1351–1362.

61. Madhi, S. A., V. Baillie, …, A. Izu. 2021. Efficacy of the ChAdOx1 nCoV-19 Covid-19 Vaccine against the B.1.351 Variant. *N. Engl. J. Med.* 384:1885–1898.
62. Mahase, E. 2021. Covid-19: Where are we on vaccines and variants? *BMJ.* 372:n597.
63. Torjesen, I. 2021. Covid-19: Omicron may be more transmissible than other variants and partly resistant to existing vaccines, scientists fear. *BMJ.* 375:n2943.
64. Ford, C. T., D. Jacob Machado, and D. A. Janies. 2022. Predictions of the SARS-CoV-2 Omicron Variant (B.1.1.529) Spike Protein Receptor-Binding Domain Structure and Neutralizing Antibody Interactions. *Front. Virol.* 2. https://doi.org/10.3389/fviro.2022.830202.
65. Golcuk, M., A. Yildiz, and M. Gur. 2021. The Omicron Variant Increases the Interactions of SARS-CoV-2 Spike Glycoprotein with ACE2. Preprint at BioRxiv. https://doi.org/10.1101/2021.12.06.471377.
66. Kumar, S., T. S. Thambiraja, …, G. Subramaniam. 2021. Omicron and Delta variant of SARS-CoV-2: A comparative computational study of spike protein. *J. Med. Virol.* 94:1641–1649.
67. Leon, P. E., T. J. Wohlbold, …, G. S. Tan. 2017. Generation of Escape Variants of Neutralizing Influenza Virus Monoclonal Antibodies. *J. Vis. Exp.* 56067. https://doi.org/10.3791/56067.
68. Xue, L. C., D. Dobbs, …, V. Honavar. 2015. Computational prediction of protein interfaces: A review of data driven methods. *FEBS Lett.* 589:3516–3526.
69. Yuan, C., H. Chen, and D. Kihara. 2012. Effective inter-residue contact definitions for accurate protein fold recognition. *BMC Bioinf.* 13:292.
70. Saha, S., and G. P. S. Raghava. 2006. Prediction of continuous B-cell epitopes in an antigen using recurrent neural network. *Proteins.* 65:40–48.
71. Saha, S., and G. P. S. Raghava. 2004. BcePred: Prediction of Continuous B-Cell Epitopes in Antigenic Sequences Using Physico-chemical Properties. *In* Artificial Immune Systems. G. Nicosia, V. Cutello, P. J. Bentley, and J. Timmis, eds Springer, Berlin, Heidelberg, pp. 197–204.
72. Yao, B., D. Zheng, …, C. Zhang. 2013. Conformational B-cell epitope prediction on antigen protein structures: a review of current algorithms and comparison with common binding site prediction methods. *PLoS One.* 8:e62249.
73. Sun, P., H. Ju, …, Y. Li. 2013. Bioinformatics resources and tools for conformational B-cell epitope prediction. *Comput. Math. Methods Med.* 2013:943636.
74. Jespersen, M. C., B. Peters, …, P. Marcatili. 2017. BepiPred-2.0: improving sequence-based B-cell epitope prediction using conformational epitopes. *Nucleic Acids Res.* 45:W24–W29.
75. Liang, S., D. Zheng, …, C. Zhang. 2010. EPSVR and EPMeta: prediction of antigenic epitopes using support vector regression and multiple server results. *BMC Bioinf.* 11:381.
76. Sweredoski, M. J., and P. Baldi. 2009. COBEpro: a novel system for predicting continuous B-cell epitopes. *Protein Eng. Des. Sel. PEDS.* 22:113–120.
77. Negi, S. S., and W. Braun. 2009. Automated Detection of Conformational Epitopes Using Phage Display Peptide Sequences. *Bioinforma. Biol. Insights.* 3:71–81.

Biophysical Reports, Volume *2*

Supplemental information

Identifying vaccine escape sites via statistical comparisons of short-term molecular dynamics

Madhusudan Rajendran, Maureen C. Ferran, and Gregory A. Babbitt

A

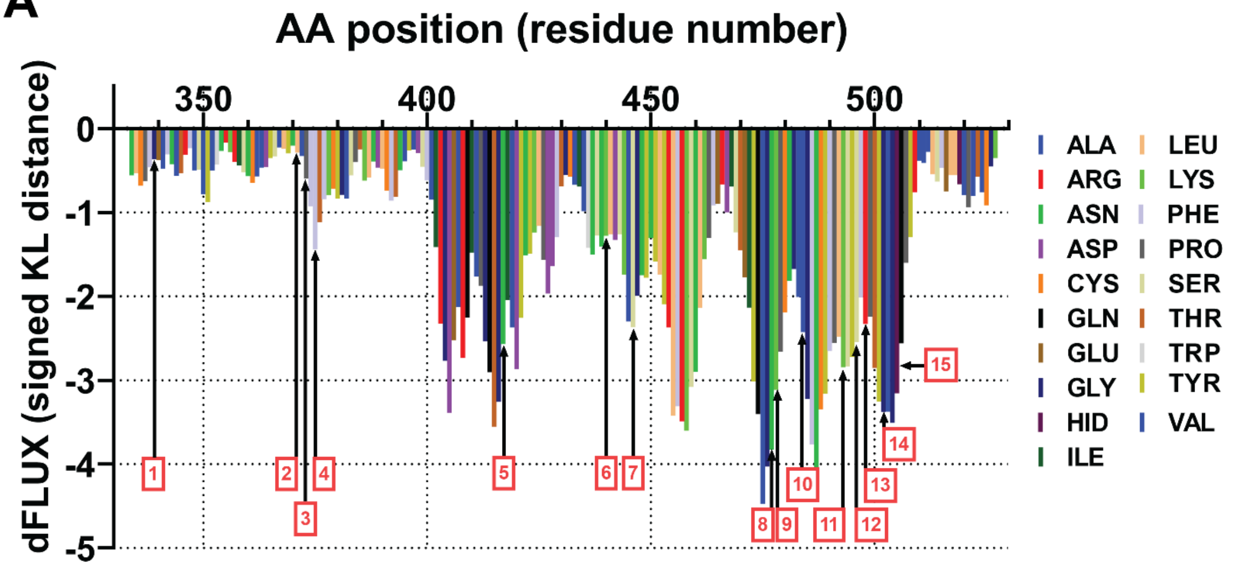

B

PDB ID	Antigen	Antibody	AA and position	-KL divergence	D value	p value
**7NXA**	Omicron RBD	COVOX-222	K417N (5)	-2.60	0.631	p < 0.001
			S477N (8)	-3.83	0.762	p < 0.001
			Q493K (11)	-2.84	0.762	p < 0.001
			N501Y (14)	-3.25	0.737	p < 0.001
			Y505H (15)	-3.16	0.771	p < 0.001

Supplemental Figure 1: Molecular dynamic simulation of the omicron variant. (A) Sequence positional plotting of dampening of atom motion on omicron RBD domain by COVOX-222. Mutation sites correspond to the Omicron RBD are labeled in red (1-15) and are listed in Figure 4B. Table summarizing the protein structure used for primary models for analyzing the molecular dynamics of COVOX-222 interaction with Omicron RBD. The table also includes amino acid positions, the corresponding -KL divergence value denoting atomic fluctuations dampening, D value and the corresponding level of significance for the KL divergence values.

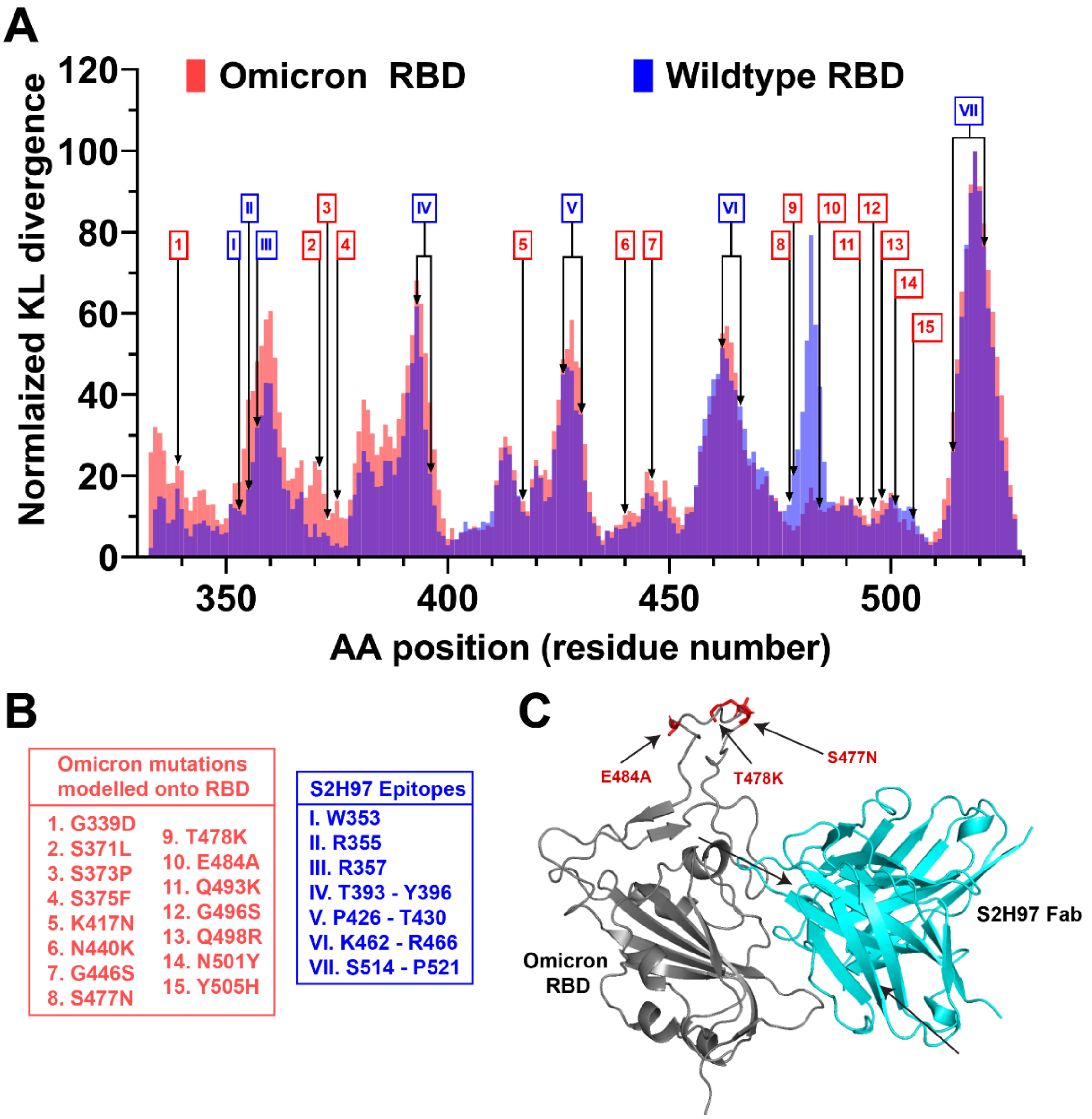

Supplemental Figure 2: MD simulations with the Omicron variant reveal sites that promote similiar binding affinity to hACE2. (A) Sequence positional plotting of the normalized dampening of atom motions on the Omicron RBD (red) and the wildtype RBD (blue) by monoclonal antibody S2H97.. The omicron RBD mutations are labeled in red (1-15). The sites corresponding to the S2H97 epitope on the wildtype RBD on labeled in blue (I – VII). The amino acid residues that correspond to the numbers and roman numerals are listed in (B). In the omicron RBD, we see alteration in atomic fluctuation peaks at several of the epitope sites.. (B) List of omicron mutations modeled onto RBD, and the list of S2H97 epitopes. (C) Crystal structure of S2H97 Fab (cyan) superimposed onto the structure of the Omicron RBD (grey) (PDB 7M7W). Some of the amino acid residues that correspond to the S2H97 epitopes, show an increase in atomic fluctuation dampening, are shown in blue. Several of the amino acid residue that correspond to omicron mutations with increased atomic fluctuation dampening, which are sites of ACE2 interactions, are shown in red. NOTE: Major changes occurring E4848A, T478K, S477N.

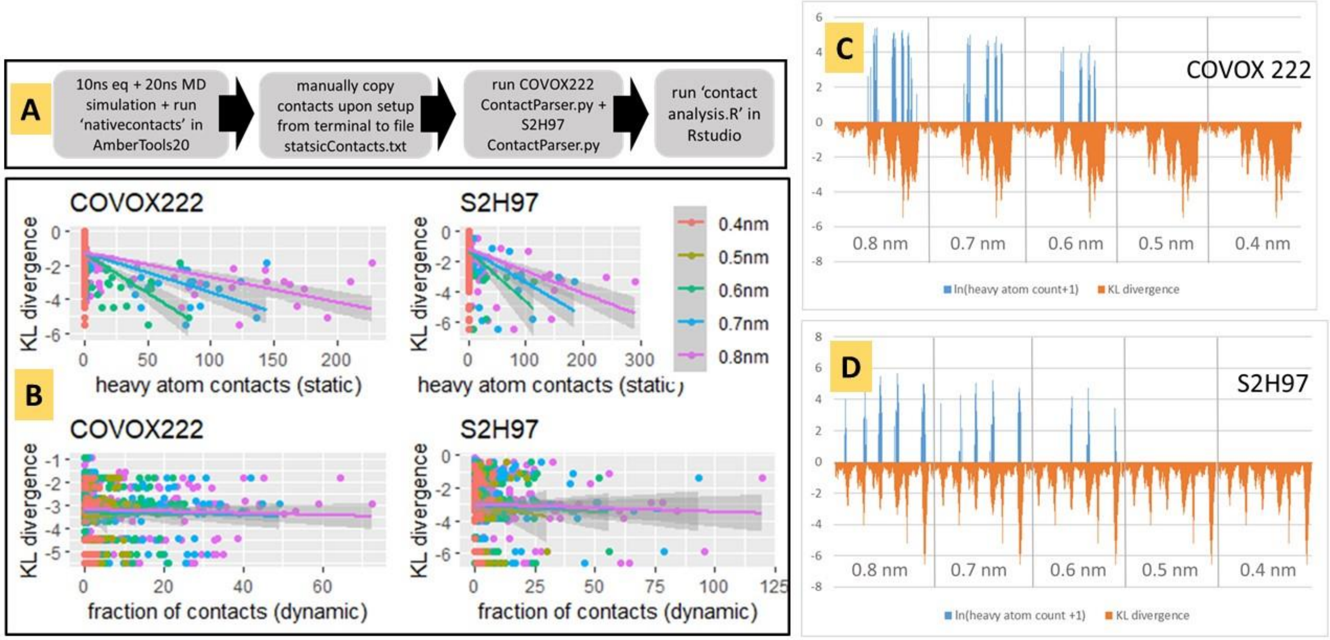

Supplemental Figure 3: Correlation between counts of heavy atom contacts between protein sites in the interface and atom dampening of sites during binding (i.e. KL divergence). The trends are shown for all interface sites for both COVOX-222 and S2H97 that fall within 4, 5, 6, 7, and 8 angstroms proximity range. Heavy atom contacts were counted in both static and dynamic conditions. For dynamic counts 100000 frames were generated from 20ns of molecular dynamics simulations on the antibody-bound viral receptor binding domain. For static counts only the first frame was used. All supporting data and code are in the S3 folder in our supplemental data file (data_RajendranFerranBabbitt_2022) (A) The analysis pipeline begins with the 'nativecontacts' program in AmberTools20 (see README.txt for the bash commands used) and proceeds subsequently via our python and R scripts (COVOX222_ContactsParser.py, S2H97_ContactParser.py, contact_analysis.R). (B) The associations between static and dynamic contact counts and the KL divergence are shown for each (See also Supplemental Table 1 below). (C-D) The respective positional plots of heavy atom contact counts and KL divergence are given for cutoff distance values of 0.4 nm – 0.8 nm. NOTE: the KL divergence mapping does not change with cutoff value and the heavy atom contact counts on the plots are scaled to ln(count+1) to allow visual comparison.

Supplemental Table 1. Correlation analyses for heavy atom contact counts and atom motion dampening (i.e. KL divergence) in SARS-CoV-2 antibody bound structures and molecular dynamic simulations of COVOX 222 and S2H97.

Correlation COVOX222	r (static)	t (static)	p (static)	r (dynamic)	t (dynamic)	p (dynamic)
0.8 nm	-0.5182787	-8.4192	8.48e-15	-0.0395004	-0.97635	0.3293
0.7 nm	-0.496611	-7.9486	1.541e-13	-0.039234	-0.79018	0.4299
0.6 nm	-0.458811	-7.1736	1.524e-11	-0.025573	-0.43788	0.6618
0.5 nm	NA	NA	NA	0.061458	0.60956	0.5436
0.4 nm	NA	NA	NA	0.0045329	0.044874	0.9643

correlation S2H97	r (static)	t (static)	p (static)	r (dynamic)	t (dynamic)	p (dynamic)
0.8 nm	-0.4745286	-7.5473	1.635e-12	-0.044418	-0.89479	0.3714
0.7 nm	-0.4501416	-7.0574	2.861e-11	-0.030993	-0.53976	0.5898
0.6 nm	-0.2893524	-4.232	3.556e-05	-0.052133	-0.74012	0.4601
0.5 nm	NA	NA	NA	-0.079197	-0.78648	0.4335
0.4 nm	NA	NA	NA	0.093881	0.9335	0.3529

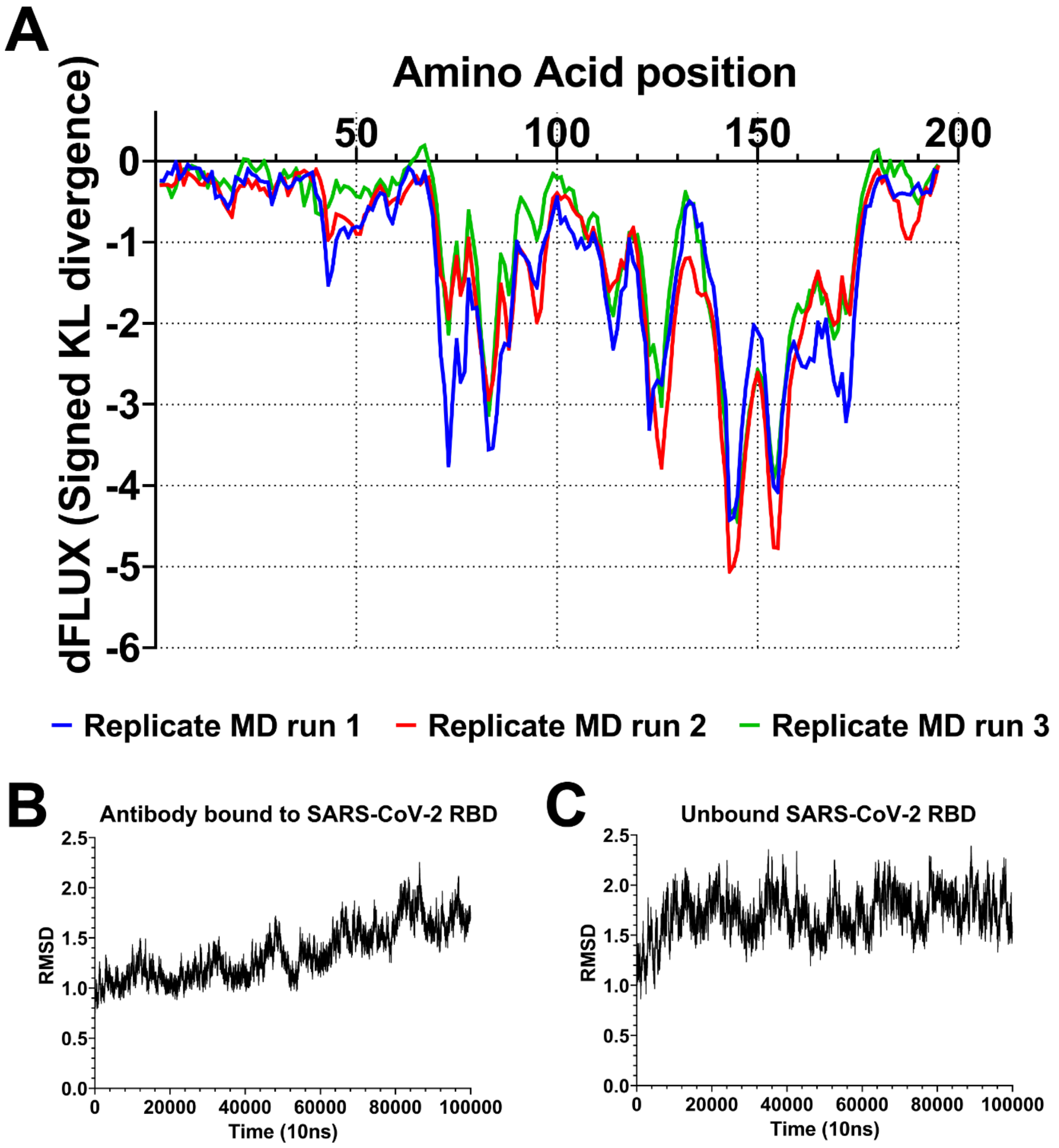

Supplemental Figure 4: Reproducibility/stability of the molecular dynamics (A) Three replicate binding signature profiles (i.e. negative KL divergence) demonstrating the reproducibility for identically initiated comparative molecular dynamics analysis of COVOX-222 bound SARS-CoV-2. Characteristic RMSD plots for the 10ns equilibration of the (B) antibody-bound and (C) unbound SARS-CoV-2 is also shown.
